# Real-time coronary artery stenosis detection based on modern neural networks

**DOI:** 10.1038/s41598-021-87174-2

**Published:** 2021-04-07

**Authors:** Viacheslav V. Danilov, Kirill Yu. Klyshnikov, Olga M. Gerget, Anton G. Kutikhin, Vladimir I. Ganyukov, Alejandro F. Frangi, Evgeny A. Ovcharenko

**Affiliations:** 1 Tomsk Polytechnic University, Tomsk, Russia; 2 Research Institute for Complex Issues of Cardiovascular Diseases, Kemerovo, Russia; 3 University of Leeds, Leeds, UK

**Keywords:** Interventional cardiology, Outcomes research, Applied mathematics, Computational science, Computer science

## Abstract

Invasive coronary angiography remains the gold standard for diagnosing coronary artery disease, which may be complicated by both, patient-specific anatomy and image quality. Deep learning techniques aimed at detecting coronary artery stenoses may facilitate the diagnosis. However, previous studies have failed to achieve superior accuracy and performance for real-time labeling. Our study is aimed at confirming the feasibility of real-time coronary artery stenosis detection using deep learning methods. To reach this goal we trained and tested eight promising detectors based on different neural network architectures (MobileNet, ResNet-50, ResNet-101, Inception ResNet, NASNet) using clinical angiography data of 100 patients. Three neural networks have demonstrated superior results. The network based on Faster-RCNN Inception ResNet V2 is the most accurate and it achieved the mean Average Precision of 0.95, F1-score 0.96 and the slowest prediction rate of 3 fps on the validation subset. The relatively lightweight SSD MobileNet V2 network proved itself as the fastest one with a low mAP of 0.83, F1-score of 0.80 and a mean prediction rate of 38 fps. The model based on RFCN ResNet-101 V2 has demonstrated an optimal accuracy-to-speed ratio. Its mAP makes up 0.94, F1-score 0.96 while the prediction speed is 10 fps. The resultant performance-accuracy balance of the modern neural networks has confirmed the feasibility of real-time coronary artery stenosis detection supporting the decision-making process of the Heart Team interpreting coronary angiography findings.

## Introduction

Coronary artery disease (CAD) is the leading cause of death worldwide^1^, affecting over 120 million people^2^. The main cause of CAD is atherosclerotic plaque accumulation^3^ in the epicardial arteries leading to a mismatch between myocardial oxygen supply and myocardial oxygen demand and commonly resulting in ischemia. Chest pain is the most likely symptom that occurs during physical and/or emotional stress, relieved promptly with rest or by taking nitroglycerin. This process can be modified by lifestyle adjustments, pharmacological therapies, and invasive interventions designed to achieve disease stabilization or regression^4^. Despite novel imaging modalities (e.g. coronary CT angiography) have been developed, invasive coronary angiography is the preferred diagnostic tool to assess the extent and severity of complex coronary artery disease according to the 2019 guidelines of the European Society of Cardiology^5,6^. Multivessel coronary artery disease affecting two or more coronary arteries requires interpretive expertise on the assessment of multiple parameters (the number of affected major coronary arteries, the location of lesions, the severity of stenosis, the length of the stenotic segment, tortuosity, etc.) during an intervention. The process of interpreting complex coronary vasculature, image noise, low contrast vessels, and non-uniform illumination is time-consuming^7^, thereby posing certain challenges to the operator. Real-time automatic CAD detection and labeling may overcome the abovementioned difficulties by supporting the decision-making process.

A number of approaches for automatic or semi-automatic assessment of coronary artery diseases have been proposed by different research groups. These approaches follow the general scheme: (1) coronary artery tree extraction, (2) calculation of geometric dimensioning, and (3) analysis of the stenotic segment. The key stage that determines the speed and accuracy of such algorithms is based on the coronary artery tree extraction using the centerline extraction^8,9^; the graph-based method^10–12^; superpixel mapping^13,14^; and machine/deep learning^15–17^. The last, being a powerful tool for computer vision and image classification, has shown great promise in CAD detection due to their performance, tuning flexibility, and optimization. The ultimate purpose that CNN developers and users are trying to meet is to strike an optimal balance between accuracy and speed, the so-called speed/accuracy trade-off^18^. While some CNNs with high performance and optimal accuracy suitable for real-time segmentation can be used on mobile devices and low-end PCs, others with low performance are highly efficient for object detection (precision, recall, F1-score, mAP). Depending upon the task complexity and scope, this balance may vary and be achieved using the proper CNN architecture. The speed/accuracy trade-off for CAD detection should be adjusted to both, elective and urgent diagnosis. On the one hand, neural networks used for determining the severity of atherosclerotic lesions should possess superior detection rate as their decision-making ability will specify the selection of treatment strategies, including life-saving procedures. This situation is typical for stable patients undergoing elective coronary angiography. Therefore, heavy-weight CNNs requiring time to process angiographic data accurately can be applied. On the other, CNNs should ensure the highest performance of real-time image processing for urgent patients who do not have time for prolonged preoperative management and should undergo percutaneous coronary intervention (PCI) immediately following the diagnostic catheterization (ad-hoc PCI)^19,20^.

Albeit several CNN-based approaches focused on achieving optimal accuracy for CAD detection with the Dice Similarity Coefficient of more than 0.75^12,13^ and/or the Sensitivity metric of more than 0.70^21^ have been proposed, their speed remains disregarded. Image processing time is an important indicator for the applied use of these methods that can reach 1.1–11.87 s^10^, 20 s^10,13^, and over 60 s^9^. However, this time is unacceptable for real-time CAD detection with the processing rate of 7.5–15 fps instead of the required 0.13–0.07 s per frame^22,23^. Slow data processing does not allow providing real-time support for the operator during the procedure and may be performed after diagnosis and data collection. Some researchers try to improve the performance of these algorithms by segmenting only large vessels of the coronary bed^18^. This approach allows achieving the inference time of 0.04 frames per second, but it does not take into account stenotic lesions in small branches. Another approach using convolutional neural networks to speed up the algorithm includes the extraction of individual regions of interest with stenotic sites without the entire coronary artery tree. A similar principle has been reported by Cong et al.^19^ describing the Inception V3 neural network and Hong et al.^20^ describing the M-net (improved version of U-net).

Our study presents a detailed analysis of available neural network architectures and their potential in terms of accuracy and performance to detect single-vessel disease. This approach is aimed at selecting the most efficient CNN architecture and further exploring the ways of its modification and optimization to ensure superior real-time classification potential for detecting multivessel coronary artery stenosis.

To summarize, our main contributions are as follows:

- A comparative analysis of the speed/accuracy trade-off for detecting single stenoses of the coronary arteries of specific state-of-the-art CNN architectures (N = 8).
- The use of RFCN ResNet-101 V2 as is without any modification allows achieving promising real-time performance (10 fps) without a big loss in accuracy.
- The benefits of CNNs reported in our study may be leveraged for the development of software aimed at optimizing and facilitating invasive angiography.

## Source data

Initial angiographic imaging series of one hundred patients who underwent coronary angiography using Coroscop (Siemens) and Innova (GE Healthcare) at the Research Institute for Complex Problems of Cardiovascular Diseases (Kemerovo, Russia) were retrospectively enrolled in the study (Table 1). All patients had angiographically and/or functionally confirmed one-vessel coronary artery disease (≥70% diameter stenosis (by QCA (quantitative coronary analysis) or 50–69% with FFR (fractional flow reserve) ≤0.80 or stress echocardiography evidence of regional ischemia). Significant coronary stenosis for the purpose of our study was defined according to 2017 US appropriate use criteria for coronary revascularization in patients with stable ischemic heart disease^21^. The study design was approved by the Local Ethics Committee of the Research Institute for Complex Issues of Cardiovascular Diseases (approval letter No. 112 issued on May 11, 2018). All participants provided written informed consent to participate in the study. Coronary angiography was performed by the single operator according to the indications and recommendations stated in the 2018 ESC/EACTS Guidelines on myocardial revascularization. The presence or absence of coronary stenosis was confirmed by the same operator using angiography imaging series according to the 2018 ESC/EACTS Guidelines on myocardial revascularization.

**Table 1:** Clinical and demographic data of the study population.

Parameter	Value
Total number of patients	100
Mean age ± SD, years	60.3 ± 13.8
Men, n (%)	68 (68%)
Women, n (%)	32 (32%)
Body mass index (kg/m^2^)	21.6 ± 5.1
Diagnosis	CAD
Class I NYHA	5 (5%)
Class II NYHA	84 (84%)
Class III NYHA	11 (11%)
**Comorbidities**	
Arterial hypertension	53 (53%)
Diabetes mellitus	14 (14%)
Chronic heart failure, classes 1–2	36 (36%)
Coronary artery stenosis > 70% (n, %)	100 (100%)

**Figure 1:**
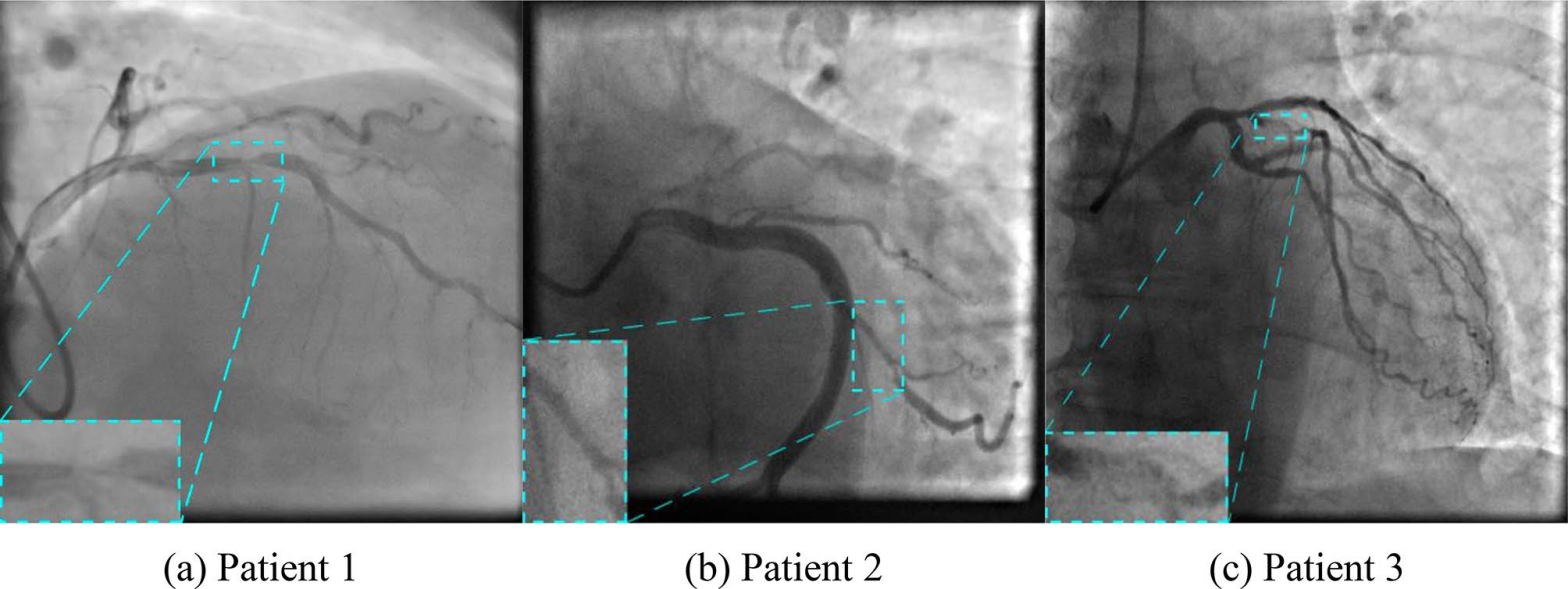
Data labelling of the source images with the callouts of the detected stenotic lesions.

Angiographic images of the radiopaque overlaid coronary arteries with stenotic segments were selected and converted into separate images. An interventional cardiologist rejected non-informative images and selected only those containing contrast passage through a stenotic vessel. A total of 8325 grayscale images (100 patients) of 512 × 512 to 1000 × 1000 pixels were included for further study. Of them, 6660 (80%), 833 (10%), and 832 (10%) images were used for training, validation, and testing respectively. In order to correctly estimate model performance, we did not randomly shuffle all 8325 images and then form data subsets. We first randomly choose patient series for the training, validation, and testing subsets in an 80:10:10 ratio, and then form those subsets. Such data split allows us to know that the validation and testing are done on the independent subsets of images and avoid bias in performance metrics. Since the training process is quite time-consuming, we excluded the usage of cross-validation for the models. Data were labeled using the LabelBox, a free version of SaaS (Software as a Service). It allows joint data labeling and subsequent validation by several specialists. Typical data labeling of the source images is shown in Fig. 1.

To analyse the source dataset, we estimated the size of the stenotic region computing the area of the bounding box. Similarly to the Common Objects in Context (COCO) dataset, we divided objects by their area into three types: small (area < 32^2^), medium (32^2^ ≤ area ≤ 96^2^), and large (area > 96^2^). 2509 small objects (30%), 5704 medium objects (69%), and 113 large objects (1%) were obtained in the input data. Since our data were unbalanced, we suppose that image analysis may be poorer on larger objects than on small and medium ones.

**Figure 2:**
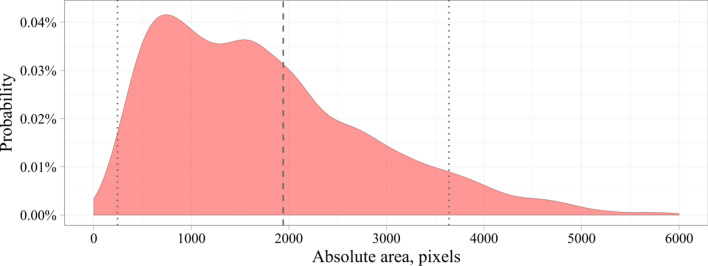
Distribution of the absolute stenotic area in the input dataset.

**Figure 3:**
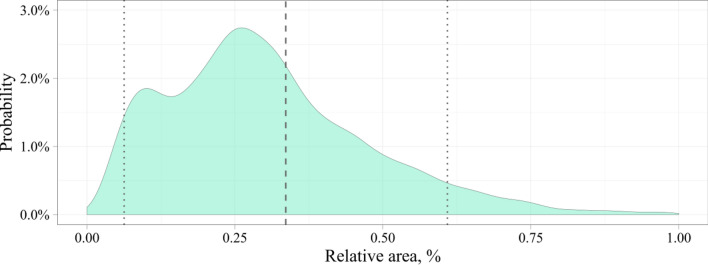
Distribution of the relative stenotic area in the input dataset.

Figures 2 and 3 show the distributions of the absolute and relative stenotic areas. To generate the distribution of the absolute area, we estimated the absolute values of the bounding box stenotic areas in pixels. To generate the distribution of the relative area, we estimated the value of the area of the bounding box relative to the area of the entire image in percentages. The dashed lines represent the mean values and standard deviations of the area. Based on the input data, the absolute stenotic area was 1942 ± 1699 pixels (Fig. 2). Since the size of the images from the input dataset varied within a certain range of values, we calculated the relative stenotic area. We selected images with normalized X and Y coordinates in the range of values [0; 1]. As a result, the relative stenotic area was 0.34 ± 0.27% (Fig. 3). As seen, the stenotic area is quite small compared to the area of the whole image that may confuse some detectors typically applied to detect objects in an unconstrained environment.

**Figure 4:**
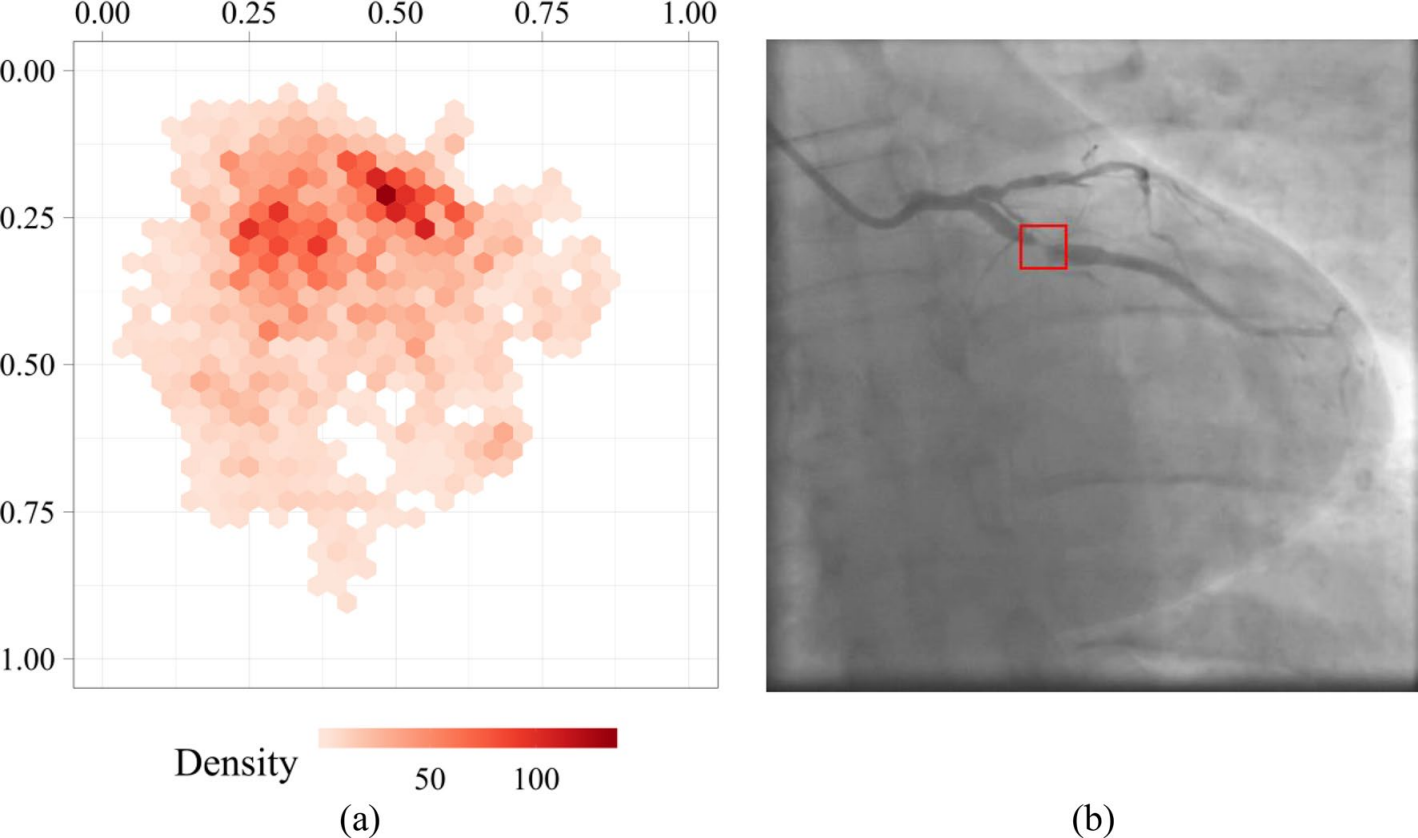
Distribution mapping of the stenosis density over the dataset (**a**) and an example of an angiography image with the labeled stenotic area (**b**).

To determine the location of stenosis accurately, we evaluated the distribution of the stenosis coordinates along the vessel in the input images. We estimated the normalized coordinates of the center point of the bounding box around the stenotic lesion. Based on this assessment, a distribution map of the coordinates of the stenosis centers was generated and is shown in Fig. 4. Each hexagon on this map reflects a number of the stenosis centers of the bounding box around the stenotic lesion. Distributing the coordinates highlights two centres with relative coordinates (0.50; 0.20) and (0.27; 0.27) along the stenotic segment. The coordinates of the stenosis centers are evenly distributed without explicit outliers. The latter should have a positive effect on training regressors based on neural networks that predict the coordinates of the bounding boxes.

## Methods

### Models description

We applied machine learning algorithms to detect coronary artery stenosis on the coronary angiography imaging series (see “Source Data” section). We examined eight models with various architectures, network complexity, and a number of weights: SSD^22^, Faster-RCNN^23^, and RFCN^24^ object detectors from the Tensorflow Detection Model Zoo^25^ based on MobileNet^26,27^, ResNet^28,29^, Inception ResNet^30^ and NASNet^31,32^. The lightweight SSD MobileNet V1 and SSD MobileNet V2 enable real-time data processing. While Faster-RCNN Inception ResNet V2 and Faster-RCNN NASNet, with over 50 million weights, were the most complex models selected for the study. Table 2 shows a brief description of the models. Characteristics of neural networks, including mAP, are reported based on their training on the COCO dataset.

**Table 2:** Brief characteristics of the use.

Model	Inference time, ms	mAP@ [0.5:0.95]	Weights, mln	Model size, Mb
SSD MobileNet V1	56	32	4.2	44
SSD MobileNet V2	31	22	6.1	19
SSD ResNet-50 V1	76	35	25.6	127
Faster-RCNN ResNet-50 V1	89	30	25.6	114
RFCN ResNet-101 V2	92	30	44.7	199
Faster-RCNN ResNet-101 V2	106	32	44.7	190
Faster-RCNN Inception ResNet V2	620	37	55.9	241
Faster-RCNN NASNet	540	–	88.9	416

### Model training

When training neural network models, their base configuration is similar to that used to train on the COCO 2017 dataset. For the unambiguous comparison of the selected models, the total number of training steps was set to 100 equal to 100′000 iterations of learning. Regarding the loss functions, the weighted Smooth L1 loss (see equation 3 in^33^) was the localization loss, and the Weighted Focal Loss was the classification loss^34^. The SSD-based models were trained using the cosine decay with the warm-up and exponential decay. When using these techniques, the learning rate gradually decreased depending on the learning step. It is also worth noting a distinctive feature of the SSD MobileNet V2 neural network, which is the use of the Hard Example Mining technique^22,35^. It allows getting additional samples of the negative class and then learns from them. Using additional samples often improves the accuracy of the stenosis location.

To train the abovementioned networks, we used models pre-trained on the COCO 2017 dataset. Using Amazon SageMaker, we tuned given models and found their best versions through a series of training jobs run on the collected dataset. Having performed hyperparameter tuning based on Bayesian optimization strategy, a set of hyperparameter values for the best performing models was found, as measured by a validation mAP. Since the network architectures significantly vary and include many parameters, we summarize the main characteristics of the training in Table 3. To train models, we used P2 (Nvidia Tesla K80 12 Gb, 1.87 TFLOPS) and P3 instances (Nvidia Tesla V100 16 Gb, 7.8 TFLOPS) from Amazon Web Services. We also divided the models into 4 groups according to their complexity for further comparison.

**Table 3:** Model training settings.

Model	Input size	Augmentation	Batch size	Type of LR	LR
SSD MobileNet V1	640 × 640 × 3	Random horizontal flip. Random crop image	4	Cosine decay with warm up	0.04
SSD MobileNet V2	300 × 300 × 3	Random horizontal flip. SSD random crop	4	Exponential decay	0.004
SSD ResNet-50 V1	640 × 640 × 3	Random horizontal flip. Random crop image	2	Cosine decay with warm up	0.04
Faster-RCNN ResNet-50 V1	600 × 600 × 3	Random horizontal flip	2	Constant LR	0.0003
RFCN ResNet-101 V2	600 × 600 × 3	Random horizontal flip	1	Constant LR	0.0003
Faster-RCNN ResNet-101 V2	600 × 600 × 3	Random horizontal flip	1	Constant LR	0.0003
Faster-RCNN Inception ResNet V2	600 × 600 × 3	Random horizontal flip	1	Constant LR	0.0003
Faster-RCNN NASNet	1200 × 1200 × 3	Random horizontal flip	1	Constant LR	0.0003

Serial changes in accuracy were obtained on the validation set during the training process. Two evaluation metrics were used to compare the performance of the selected neural networks. Precision, Recall, and F1-score were used to compare the classifiers and the mAP metric was used to judge object localization^36^. For mAP a predefined threshold value for Intersection over Union equal to 0.5 was used.

**Figure 5:**
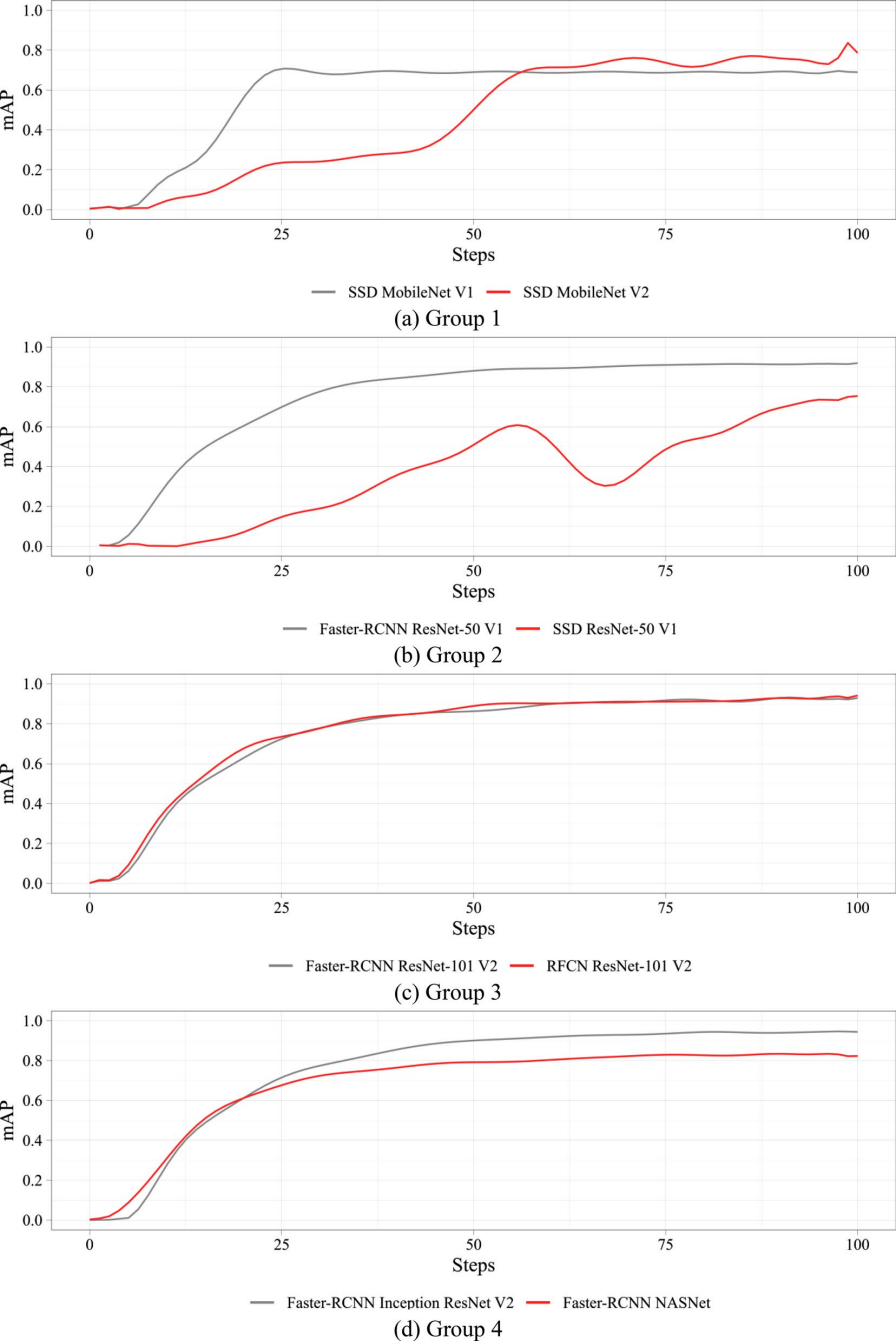
Dynamics of the mAP metric over the training process.

Figure 5 shows smooth changes in the mAP on the validation set during the training process. All models converge to a specific value of the asymptotic accuracy. SSD ResNet-50 V1 could achieve higher quality with longer training, but this would require more steps.

## Results

### Comparative assessment

**Figure 6:**
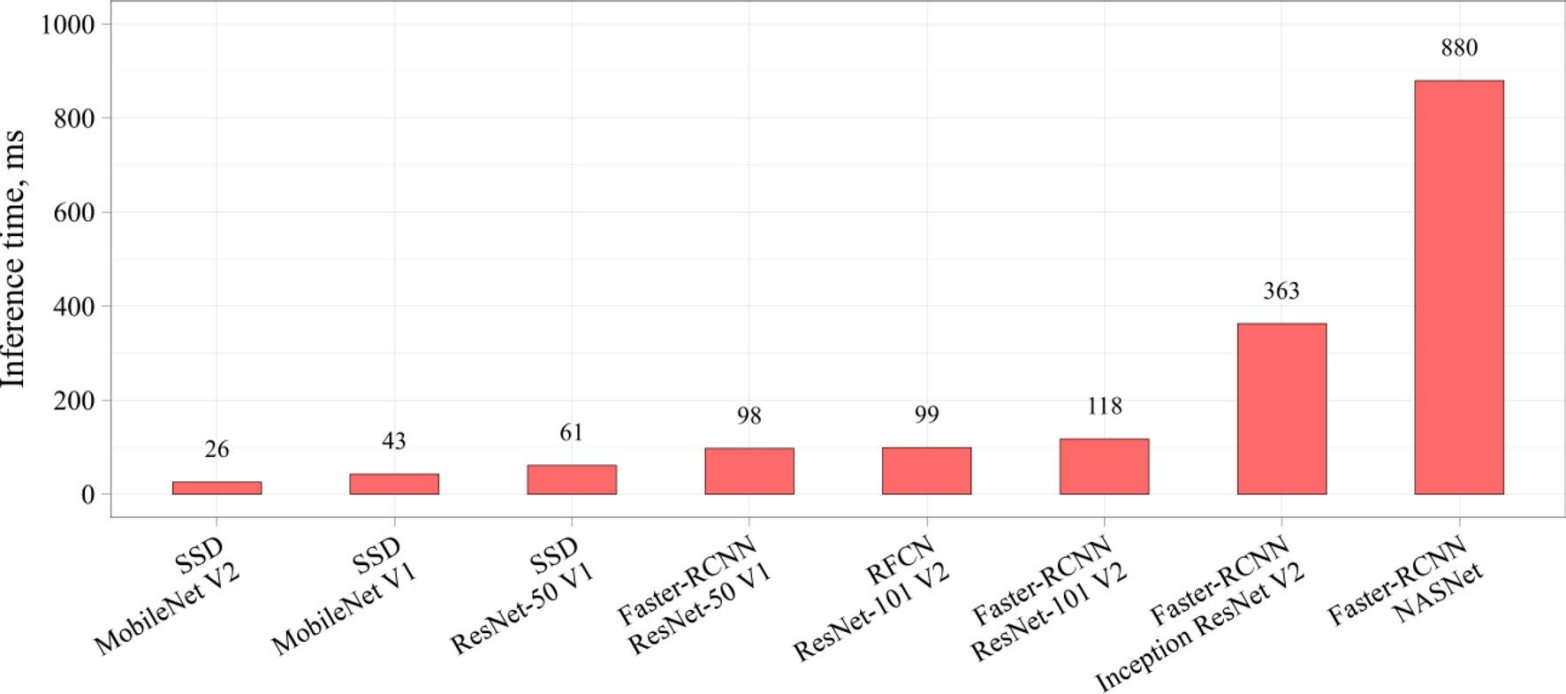
The inference time of the selected neural network models.

**Figure 7:**
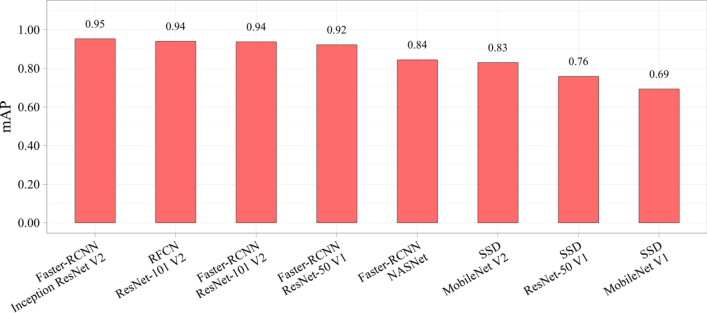
The mAP metric of the selected neural network models.

**Figure 8:**
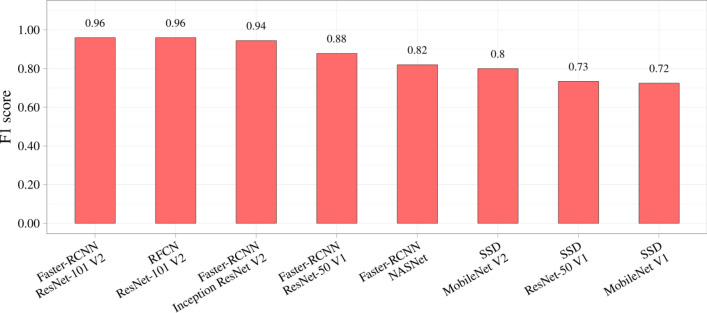
The F1 score of the selected neural network models.

Table 4 presents the results of the comparative study of the neural networks. In addition to the absolute values of the metrics, the relative values are also reported. The metrics of SSD MobileNet V1 were used as a benchmark to compare with other models. Color scale formatting reflects the distribution of models by their accuracy, training and inference times, and a number of weights, where deep blue shows the best value, and white—the worst. Figures 6, 7 and 8 show three basic metrics, the inference time, mAP, F1-score for the prediction of the stenotic lesion bounding box on an image.

**Table 4:**
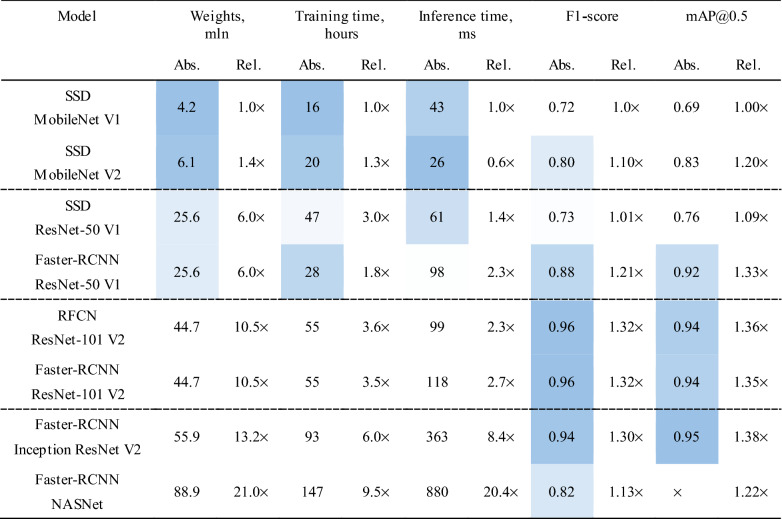
Comparative study of the selected models.

The inference time was estimated using the P3 instance (Nvidia Tesla V100 16 Gb, 7.8 TFLOPS) of Amazon Web Services. We concluded that the inference time directly depended on the complexity of the model and the total number of its weights. Thus, Faster-RCNN Inception ResNet v2 and Faster-RCNN NASNet were the slowest in predictions. Their mean processing times per one image were 363 and 880 ms, respectively. While testing the lightweight models based on the MobileNet backbone, we found that MobileNet V2 with a larger number of weights (6.1 mln) demonstrated superior inference time than Mobile Net V1 (4.2 mln). In general, MobileNet V2 had the most superior inference time than other models. Thus, it may be used for predicting the location of stenosis in real-time.

In terms of the mAP metric and F1-score, Faster-RCNN Inception ResNet V2 was the most accurate model. The mean Average Precision of this model on the validation set was 0.95, F1-score 0.96 with the inference time of 363 ms/image (≈ 3 frames per second). The fastest and relatively lightweight SSD MobileNet V2 had the mean Average Precision of 0.83, F1-score 0.80 with an inference time of 26 ms/image (≈ 38 frames per second). Based on the obtained results, we concluded that RFCN ResNet-101 V2 is an optimal one to solve the set tasks. The mAP of this model is 0.94, F1-score 0.96 and the inference time is 99 ms/image (≈ 10 frames per second). In terms of both classification (F1 score) and localization (mAP) metrics, Faster-RCNN ResNet-101 V2, RFCN ResNet-101 V2, and Faster-RCNN Inception ResNet V2 remain the most effective models for the task of stenosis detection. Additional performance metrics, such as precision and recall, are reflected in Online Appendices F and G.

### Model testing

**Figure 9:**
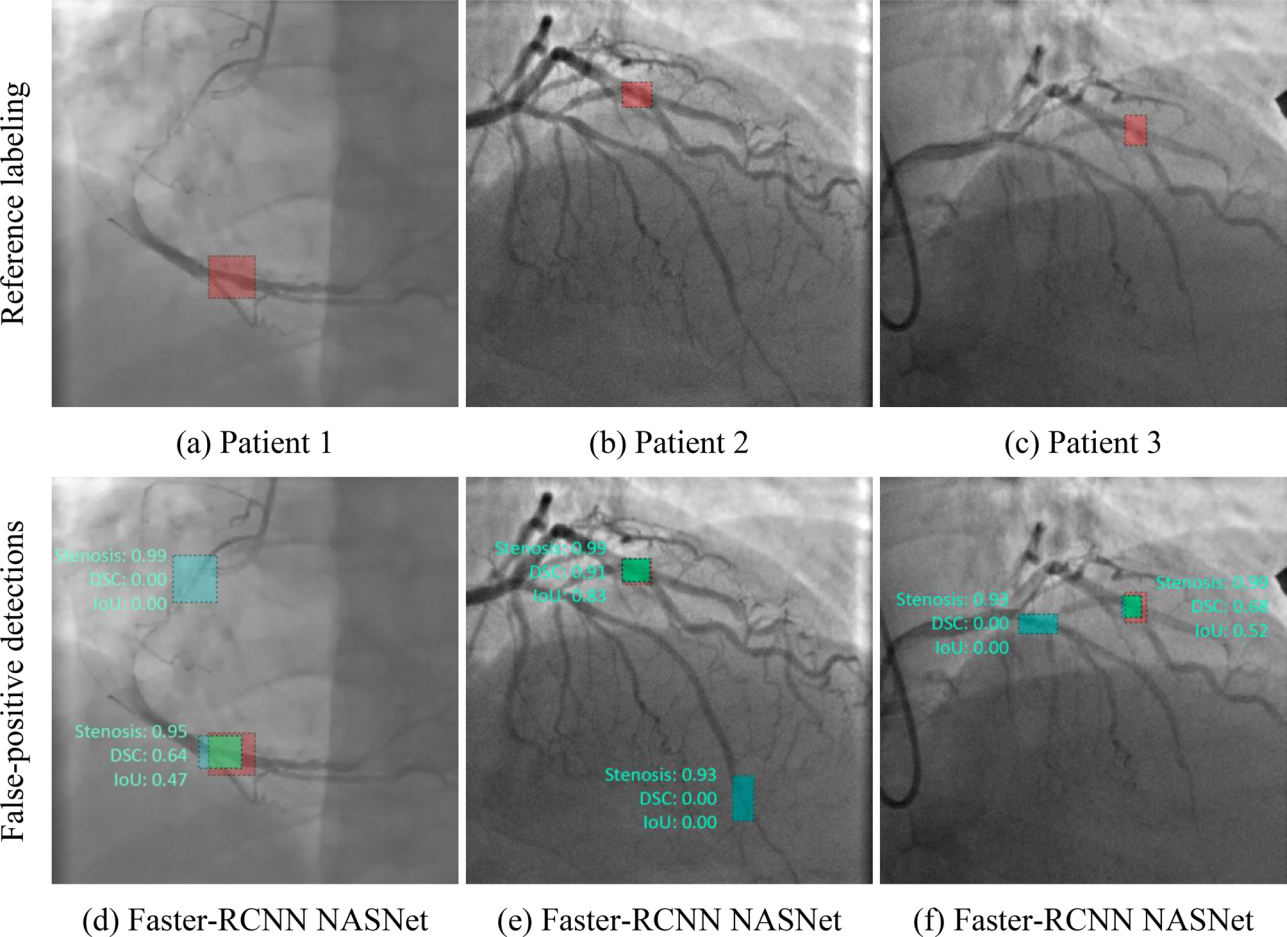
Example of the false positive predictions obtained using Faster-RCNN NASNet neural network for three test patients with the referenced labelling.

The capabilities of the selected neural networks are presented using the data of three patients with the referenced labeling (Fig. 9a–c). Detailed visualization for predictions is presented in Online Appendices H–J. The models with the best values of the loss function and mAP were used for testing.

Table 5 reports the best steps with the model optimal weights. Such localization metrics as Intersection over Union (IoU) and Dice Similarity Coefficient (DSC) were also computed and shown.

**Table 5:** Best steps with optimal model weights.

Model	Best step
SSD MobileNet V1	24
SSD MobileNet V2	99
SSD ResNet-50 V1	100
Faster-RCNN ResNet-50 V1	84
RFCN ResNet-101 V2	97
Faster-RCNN ResNet-101 V2	94
Faster-RCNN Inception ResNet V2	83
Faster-RCNN NASNet	95

**Figure 10:**
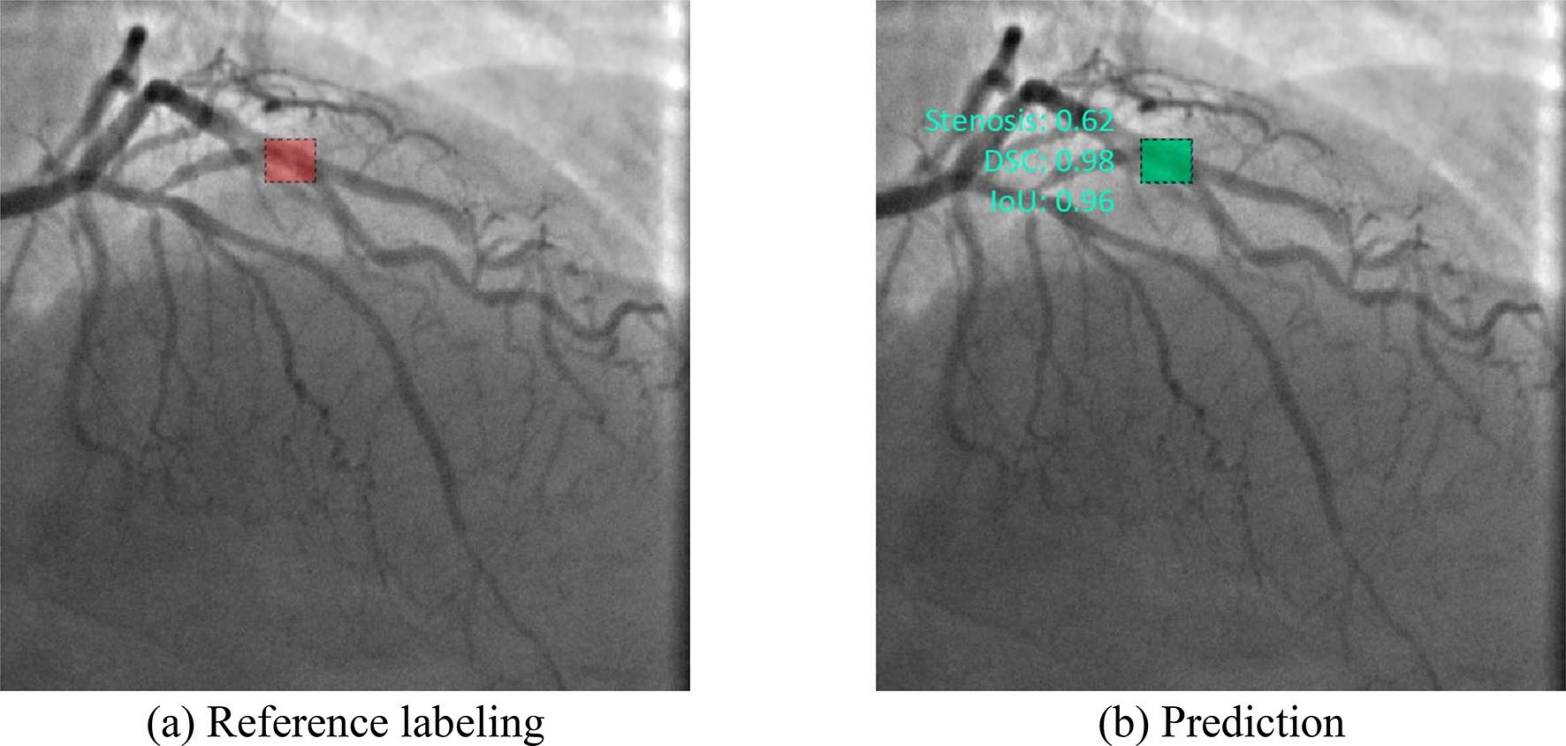
Example of the best prediction compared to reference labeling: data of patient 2 processed with SSD MobileNet V2 network.

Almost all models may accurately detect the location of stenosis. However, we faced several false positives while testing the Faster-RCNN NASNet model. In all three cases, this model detected the location of false stenotic segments with a probability of more than 90% in the right coronary artery (Fig. 9d) and the anterior descending artery (Fig. 9e, f) besides the reference stenotic region. SSD MobileNet V1 and SSD ResNet-50 V1 models failed to detect the location of stenosis in patient 1. SSD MobileNet V2 model demonstrated one of the best results in predicting the location of stenosis (Fig. 10). Despite the DSC metric of 0.65 in patient 3, it had the highest DSC metric in patients 1 and 2 (0.93 and 0.98, respectively). Additionally, the detectors based on the ResNet architecture, Faster-RCNN ResNet-50 V1 and Faster-RCNN ResNet-101 V2, should be noted. The average DSC metric on the test data was 0.85 and 0.84, respectively.

## Discussion

The ultimate goal of our study is to develop a novel stenosis detection algorithm for patients with multivessel CAD, as they represent the most difficult group for diagnosis and interpretation. We believe that automatic detection and grading of multivessel CAD may facilitate the operator work by minimizing the risk of misinterpretation and accelerate the decision-making regarding the proper treatment strategy. To date, the accuracy and certainty of interpreting coronary angiograms fully rely on the operator who needs to identify the location of the stenosis and describe individual coronary vasculature, including the diameter of the affected vessels, the length of the stenotic segments, the presence of any lateral branches, any shunts, tortuosity, etc.^37^. We have successfully tested our algorithm for detecting single-vessel CAD to assess its potential for the key task. Real-time detection of multivessel disease and its automatic grading is a more complex and multicomponent task. According to the obtained results, we concluded that the current version of our algorithm fully corresponds to the following key criteria—sufficient processing speed and detection accuracy.

### Image processing speed

From the technical point of view, the speed of the algorithm for real-time detecting coronary artery stenosis and grading its severity is one of the key parameters empowering accurate CAD diagnosis and treatment. Coronary angiography is an invasive procedure that is associated with radiologic exposure, obviating repeated contrast injections, and limiting interventional cardiologists in their manipulations. In this respect, the ability to perform real-time detection of the stenotic lesions and their simultaneous grading in the cath-lab significantly increases the diagnostic efficiency (e.g. if the algorithm is sufficiently accurate, the operator may refuse additional contrast injection and proceed with stenting). Algorithms that generate predictions slowly (inference time of 600–800 s per angiography projection) are limited in use. They should be used separately, after coronary angiography, and may serve for off-line research descriptive tasks. Since the prolonged door-to-balloon time significantly affects the patient's outcome^38^ and is directly associated with mortality^39^, the minimization of time spend on diagnosis will facilitate the decision-making process, especially for severe cases (e.g. myocardial infarction).

The existing research teams mainly focus on the accuracy of the algorithms rather than their speed. Most of them do not fit for routine medical image processing. Some of the recently reported image processing algorithms are generally perceived as slow with a high “cost” of frame analysis: Fang et al. reported the inference time varying from 1.1 to 11.87 s^10^; M’Hiri et al.—20 s^13^; and Wan et al.—63.3 s. to build the skeleton of the artery and 70.9 s. for the subsequent processing cycle^9^. Other studies have demonstrated a faster data analysis, spending almost 1.8 s per artery^17^, and 32 ± 21 s per each stenotic segment^20^. However, these algorithms use computed tomography imaging series, which are commonly obtained during routine preoperative management but not urgently. Therefore, they are spending much more time on the descriptive analysis, empowering the decision-making process. Yang et al. have recently reported the use of convolutional neural networks for segmenting major coronary arteries^18^. The algorithm spends 60 ms per angiogram, but it does not predict stenotic lesions of other small vessels.

There are no strict requirements for the processing speed of the angiography imaging series. It depends mainly on individual application settings. Thus, algorithms developed to support diagnostic angiography, performed with the aim of subsequent emergent blood flow restoration, should correspond to the following requirements: input video frame rate of 7.5–15 frames per second^40,41^, the duration of the procedure less than 25 min, and individual preferences of the operator^36^. We concluded that neural network architectures with an inference time of less than 66 ms are suitable for this task (Table 4. SSD MobileNet V1, SSD MobileNet V2, and SSD ResNet-50 V1), as they process at least 15 frames per second. However, their performance was assessed on a relatively simple case requiring detecting the location of stenosis without calculating its quantitative parameters. Thus, we expect that a detailed analysis of multivessel CAD may require a much longer time. Neural network models with the inference time of 98–118 ms per frame (Table 4. Faster-RCNN ResNet-50 V1, RFCN ResNet-101 V2, and Faster-RCNN ResNet-101 V2) may be assigned to the “grey zone”, processing 8–10 frames per second. Their resultant performance is insufficient, but they can be used in the cath-lab with the detection lag. The heavyweight models with the inference time of over 360 ms per frame (Table 4. Faster-RCNN Inception ResNet V2 and Faster-RCNN NASNet), do not fully correspond to the needs of the real-time angiography analysis, as they will fail to provide adequate productivity in complex cases.

CNN performance correlates with the complexity of their architectures. The number of weights is the foremost parameter responsible for the inference time. An increase in the number of weights has resulted in improved inference time (Table 4). Therefore, a number of CNN developers (e.g. GoogLeNet, ResNet, MobileNetV2) aim at minimizing the number of weights and size of neural networks for real-time applications, compacting them, and reducing the requirements for hardware performance^45,46^. Different approaches to these modifications have been reported, including neural network compression accelerating the inference time: tensor decomposition, quantization^47^, pruning^48^, teacher-student approaches^49^, specific layer pruning and fusions^50^, using many fewer proposals than is usual for Faster R-CNN^18^, Low-rank decomposition^51^.

### Accuracy

Detection accuracy is another important parameter indicative to the quality of the algorithm, particularly for borderline cases, when the treatment strategy is not clearly defined and false positives may mislead the Heart Team to choose a more invasive treatment option^38^. Therefore, it seems necessary to discuss these two cases separately—false positives and false negatives in the detection of stenosis. A false positive is an error in data reporting when an algorithm detects incorrectly the presence of stenosis. It may result in choosing coronary artery bypass grafting (CABG) rather than PCI since the operator relies on the misinterpreted data regarding the multiple stenotic lesions that increase individual SYNTAX Score^38,42^. Thus, we should take seriously false positives produced by the Faster-RCNN NASNet network, that misinterpreted the clinical states of three control patients (Fig. 9d–f). Alternatively, a false negative is an error in data reporting when an algorithm reports the absence of the existing stenosis. However, false negatives are less serious than false positives, as they can be leveled out during stenting by repeated contrast injection that will visualize the missed stenosis. This type of error was encountered for the two selected neural networks, the lightweight SSD MobileNet V1 and SSD ResNet-50 V1. Both these models showed the worst mAP of 0.69 and 0.76; F1-score of 0.72 and 0.73, respectively. Since these neural networks have demonstrated the worst mAP and F1-score, they are considered to be unpromising candidates for further optimization. Other models with an mAP of 0.94–0.95 and F1-score > 0.9 (Table 4) have room for further acceleration to detect multivessel CAD.

Resultant values of the classification and localization metric parameters are generally consistent with the recently published studies. Fang et al. reported an F1-score of 0.81–0.89^10^. Similar results were shown by Wan et al.^9^ and Zheik et al.^17^ equal to 0.83–0.94 and 0.75–0.88, respectively. While Yang at el. demonstrated the range of F1-score from 0.64 to 0.94^24^. Faster-RCNN InceptionResNet-v2 has been reported as the most accurate (F1-score up to 0.94) in a similar study focusing on exploring the performance of CNN architectures for detecting large arteries^24^. In our study, F1-score ranged from 0.72 to 0.96. The direct comparison of mAP values with those obtained in other studies is complicated by the different underlying performance metrics, as the Dice coefficient was reported. Therefore, we computed the Dice Similarity Coefficient that varied from 0.64 to 0.93 on the validation set and found that our data are in line with the previously reported studies: the Sensitivity metric varying from 0.59 to 0.72 in^19^, the Dice Similarity Coefficient of 0.75 in^13^ and 0.74 to 0.79 in^12^.

We found that RFCN ResNet-101 V2 neural network provides the best speed/accuracy trade-off. In addition, the task for real-time CAD detection may be progressed through its modification and hardware upgrade^18^^,47–51^. This balance may be achieved for other high-speed CNNs (SSD MobileNet V2) by improving their accuracy. Both, the accuracy and the number of errors, may potentially be improved using traditional approaches, including an increase of the training set size and its heterogeneity in addition to the use of more scalable and efficient neural network architectures (e.g. EfficientDet or CenterNet detectors^43,44^).

## Conclusion

The imbalance between accuracy and computer performance has been previously limited to the introduction of an automatic CAD detection algorithm in clinical practice. We have demonstrated that the development of hardware performance and appearance of the recent neural network architectures may significantly reduce the labor-intensive process during conventional invasive coronary angiography. We trained eight promising detectors based on different neural network architectures (MobileNet, ResNet-50, ResNet-101, Inception ResNet, NASNet) to detect the location of stenotic lesions using angiography imaging series and assessed their performance. Out of them, three neural networks have demonstrated superior results. Faster-RCNN Inception ResNet V2 is the most accurate to detect single-vessel disease. It demonstrates the mean Average Precision of 0.954, and the prediction rate of 363 ms per image (≈ 3 frames per second) on the validation set. The relatively lightweight SSD MobileNet V2 model is the fastest with an mAP of 0.830 and a mean prediction rate of 26 ms per image (≈ 38 frames per second). RFCN ResNet-101 V2 has demonstrated an optimal accuracy-to-speed ratio. Its mAP is 0.94, and the prediction speed is 99 ms per image (≈ 10 frames per second). The resultant performance-accuracy balance using the described neural networks has confirmed the feasibility of real-time CAD tracking supporting the decision-making process of the Heart Team. Real-time automatic labeling has opened new horizons for the diagnosis and treatment of complex coronary artery disease.

## Supplementary Information

Supplementary Information.
