# Supplementary material for: Real-time coronary artery stenosis detection based on modern neural networks
Source: Sci Rep. 2021 Apr 7;11:7582. doi: 10.1038/s41598-021-87174-2 (PMC8027436; doi:10.1038/s41598-021-87174-2)
Supplement: Supplementary file 1 — Supplementary Information. [file 41598_2021_87174_MOESM1_ESM.docx]

**Appendix A. Comparison of the weights of the selected neural network models**

| 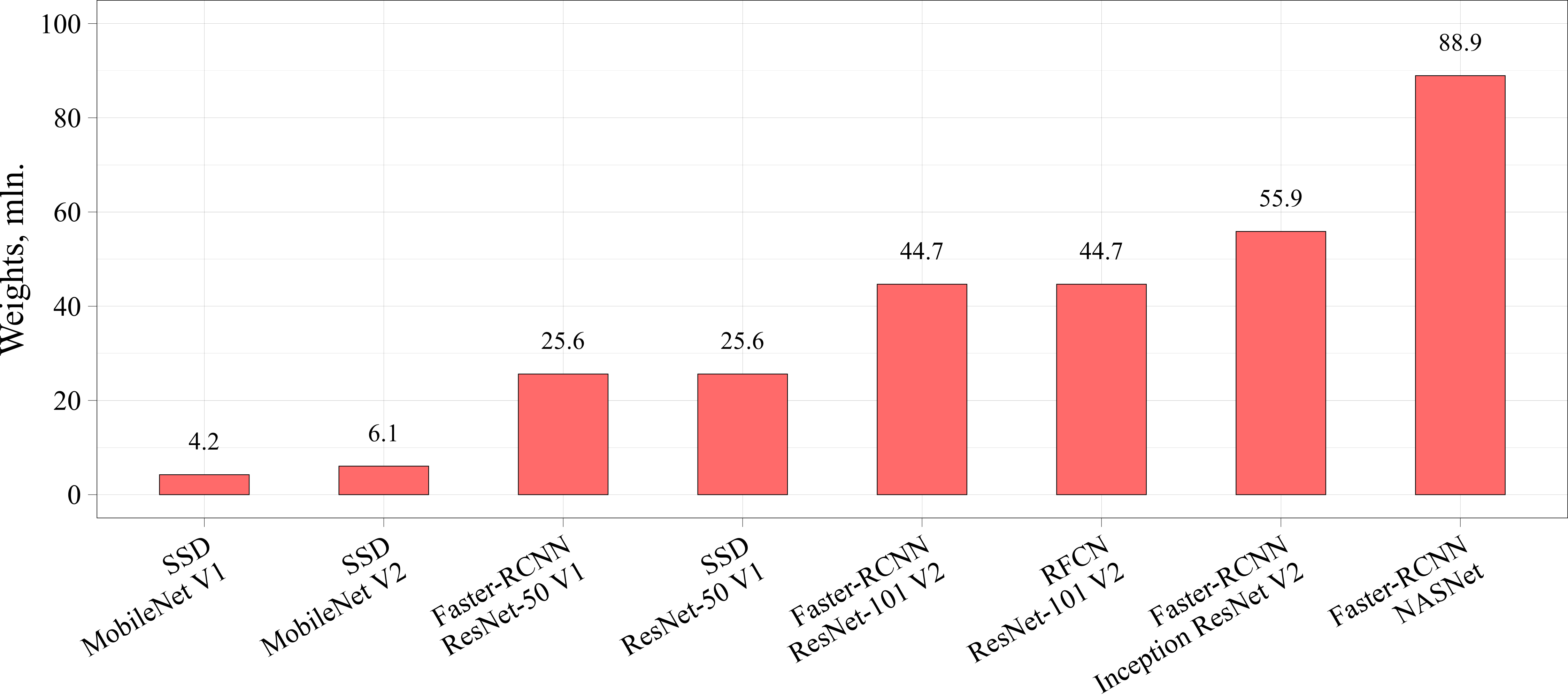 |
| --- |
| (а) Absolute number of weights |
| 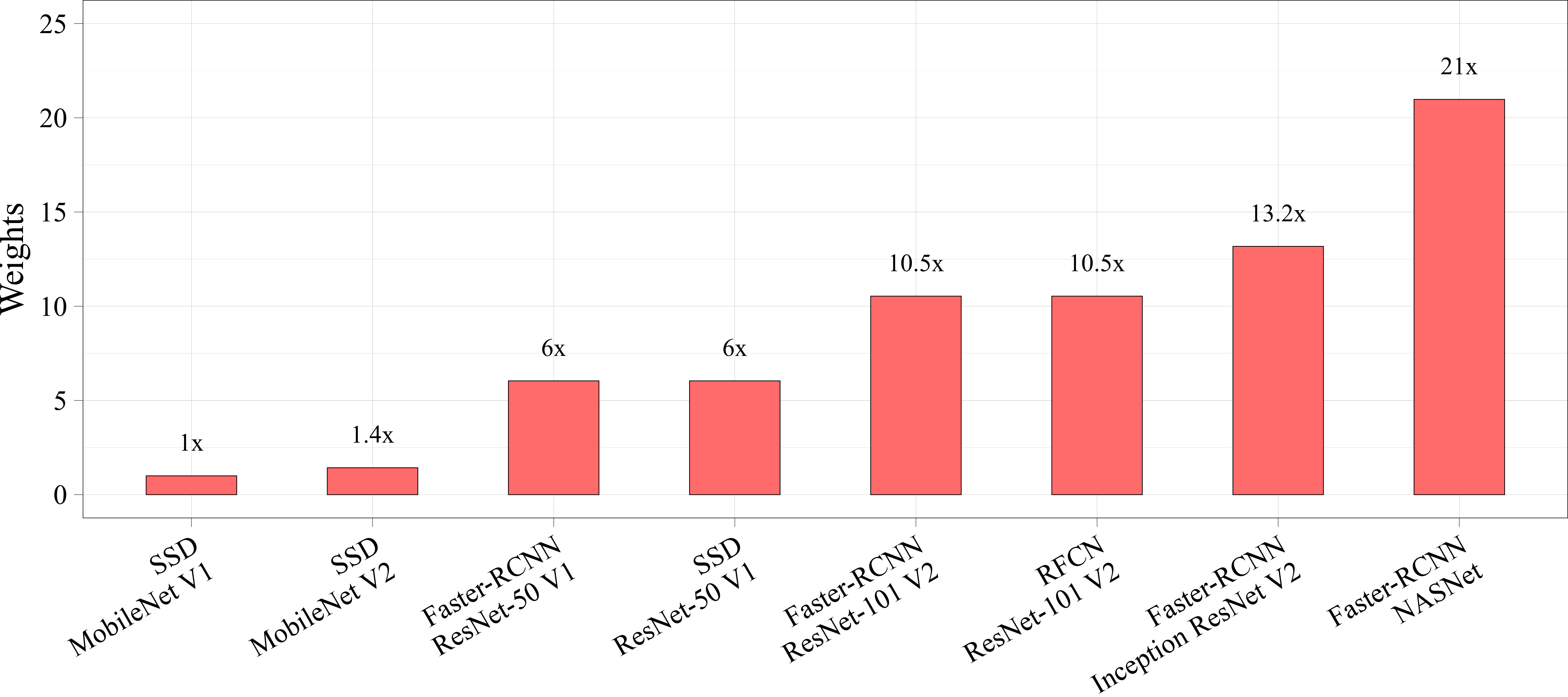 |
| (b) Relative number of weights |

**Appendix B. Comparison of the training time of the selected neural network models**

| 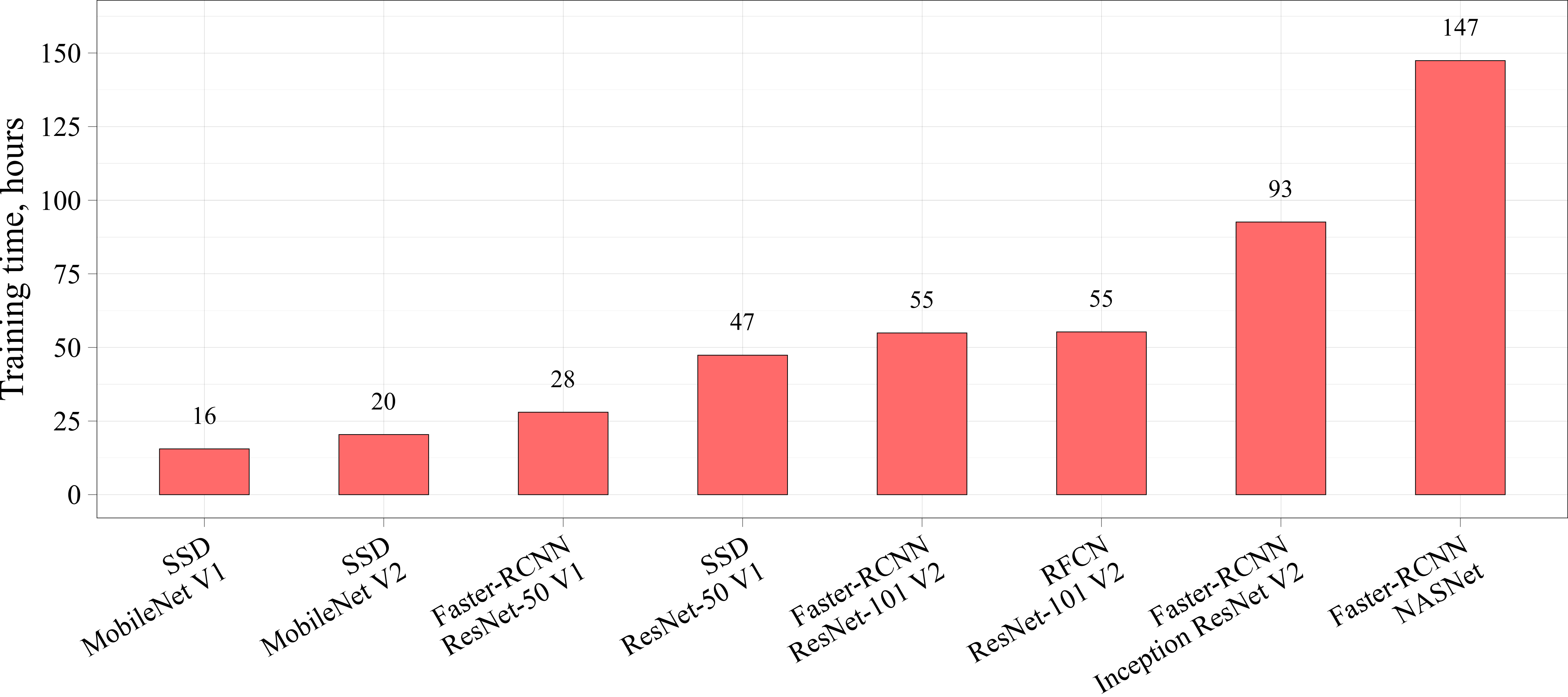 |
| --- |
| (а) Absolute values of the training time |
| 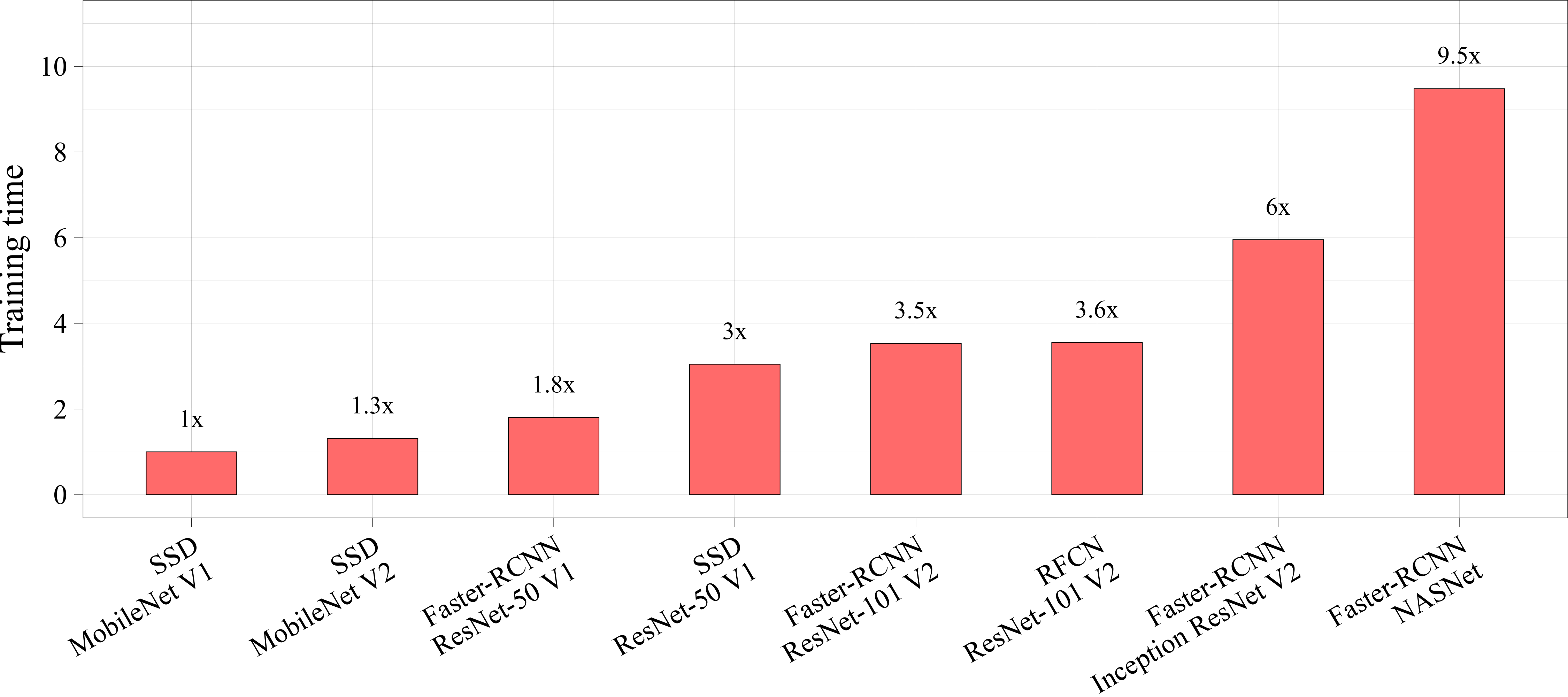 |
| (b) Relative values of the training time |

**Appendix C. Comparison of the inference time of the selected neural network models**

| 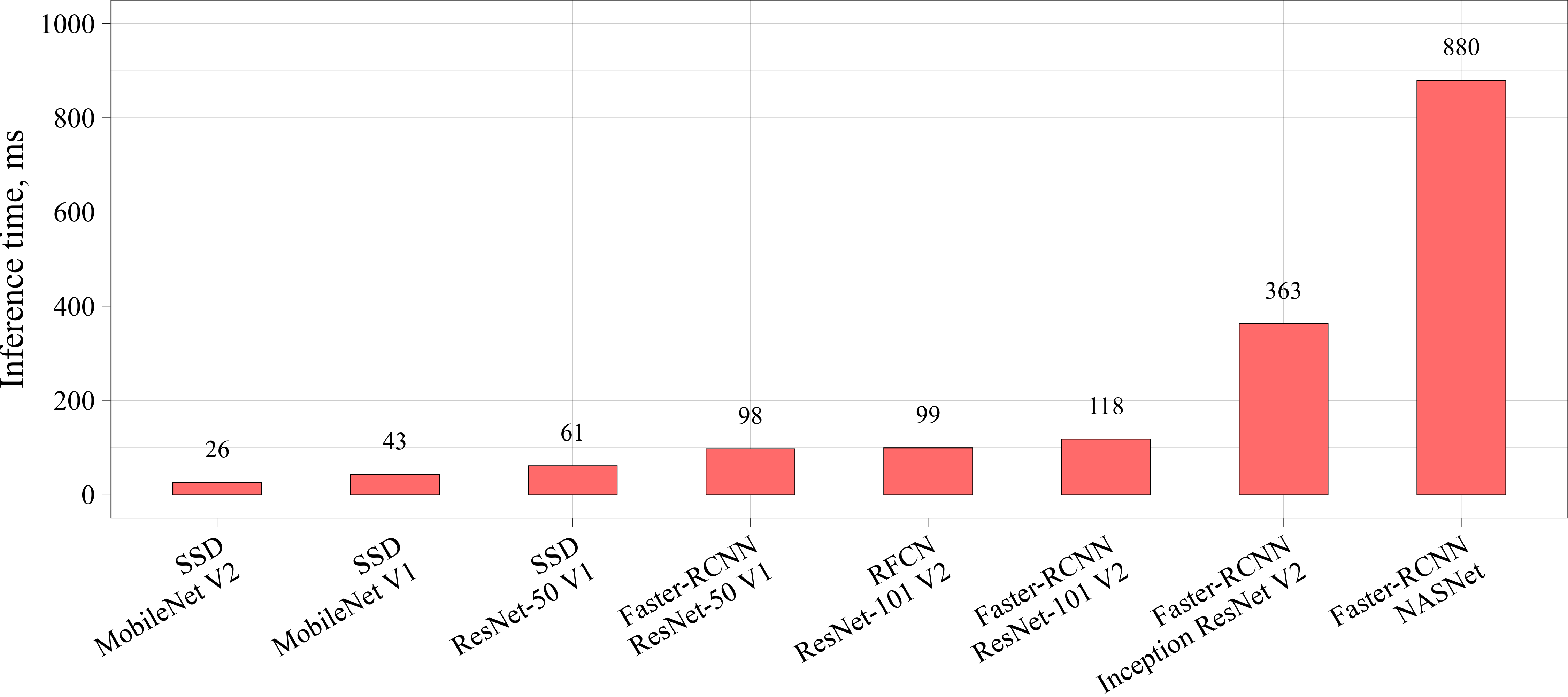 |
| --- |
| (а) Absolute values of the inference time |
| 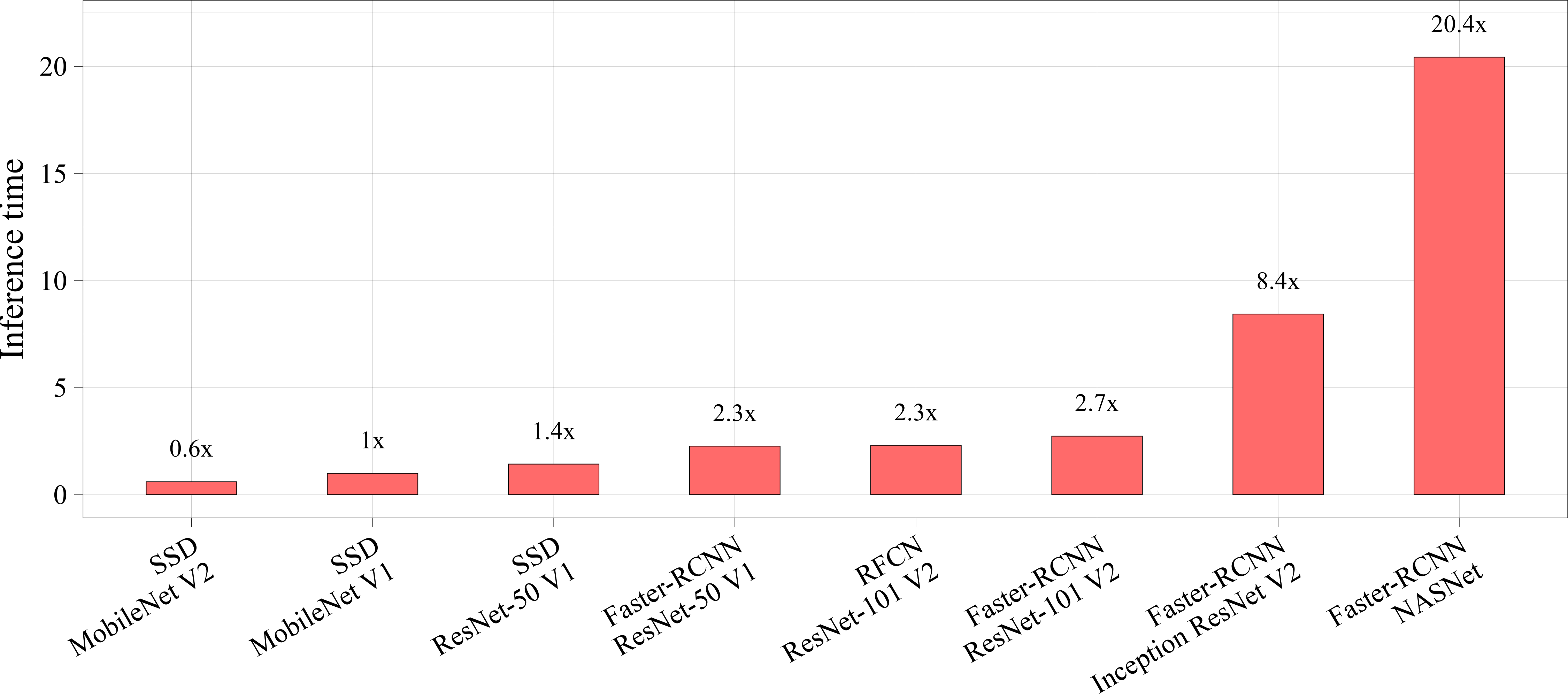 |
| (b) Relative values of the inference time |

**Appendix D. Comparison of the mAP metric of the selected neural network models**

| 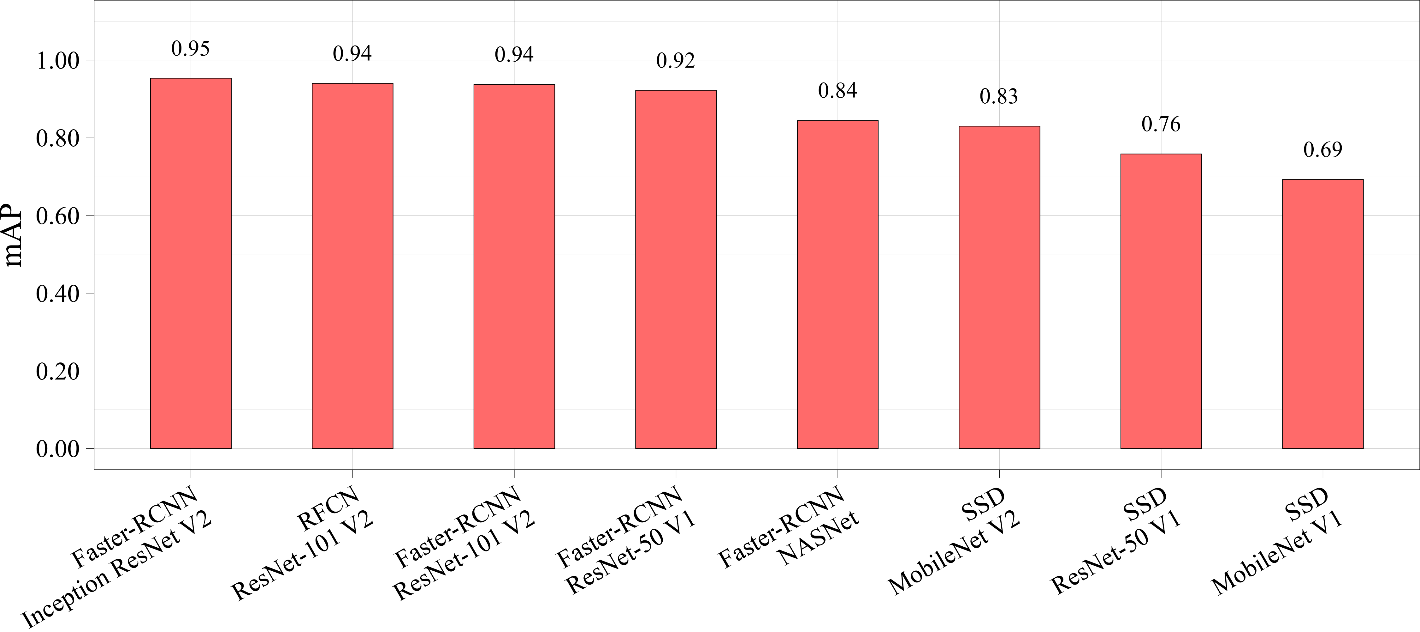 |
| --- |
| (а) Absolute values of mAP |
| 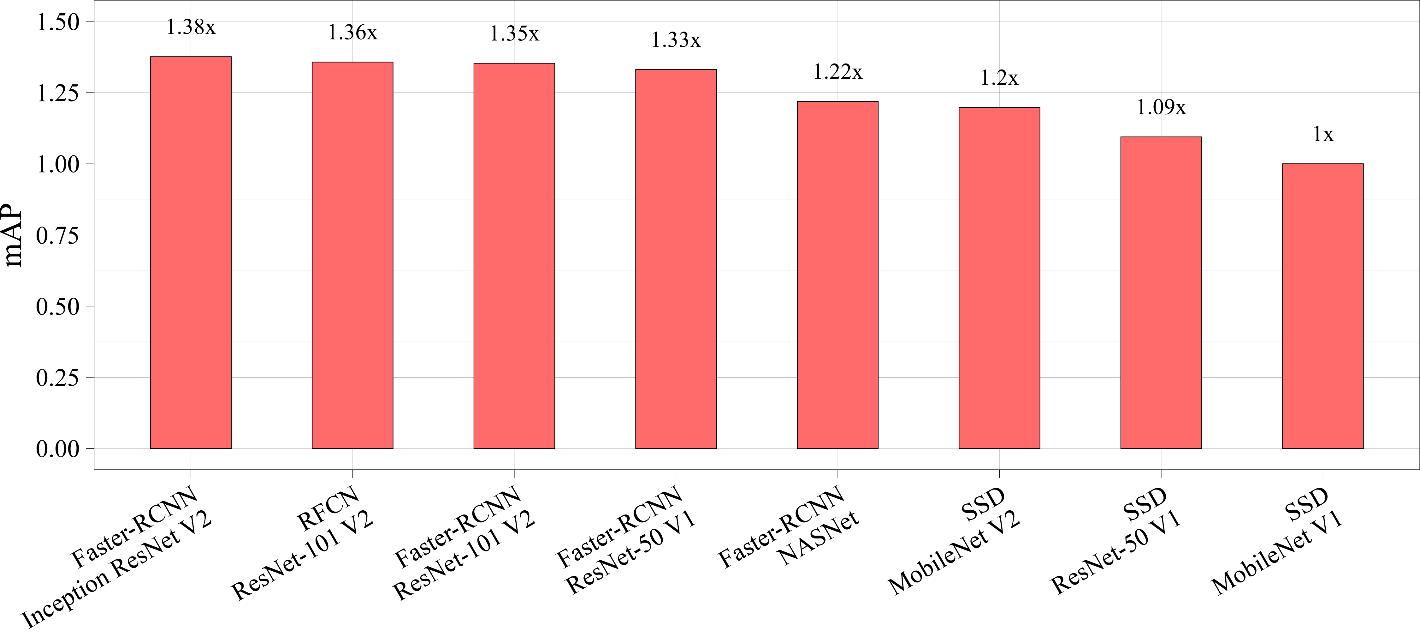 |
| (b) Relative values of mAP |

**Appendix E. Comparison of the F1 score of the selected neural network models**

| 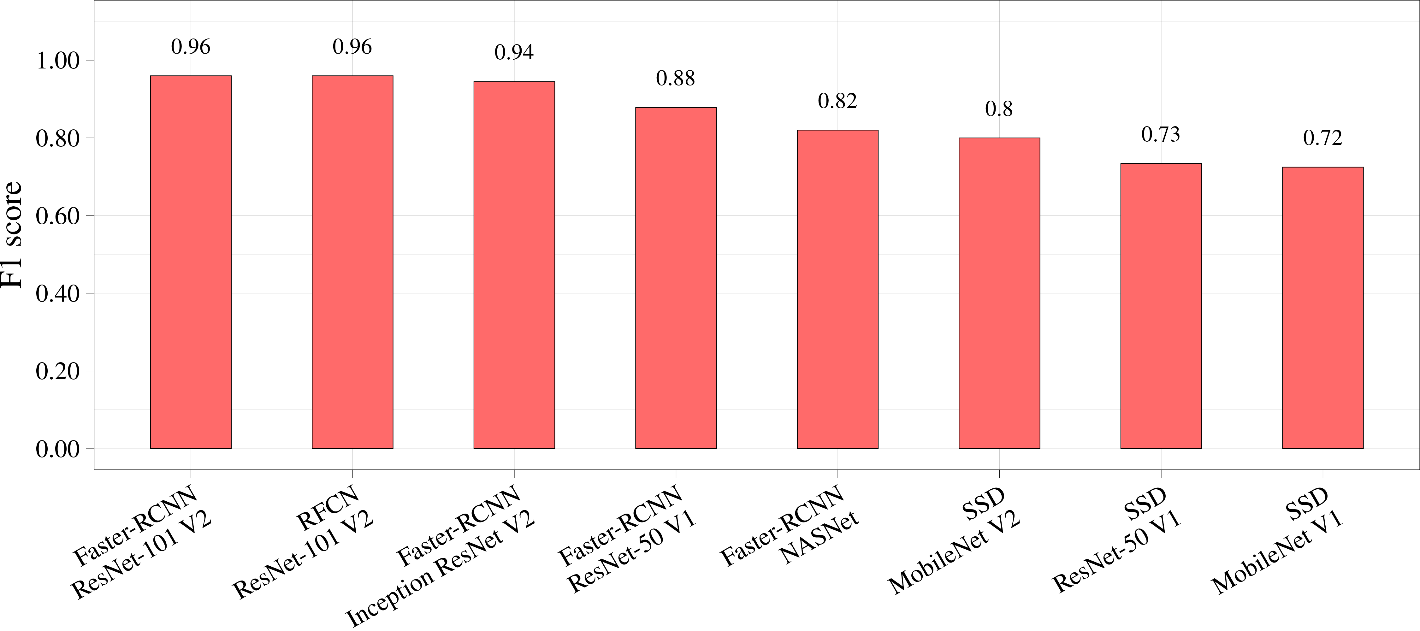 |
| --- |
| (а) Absolute values of F1 score |
| 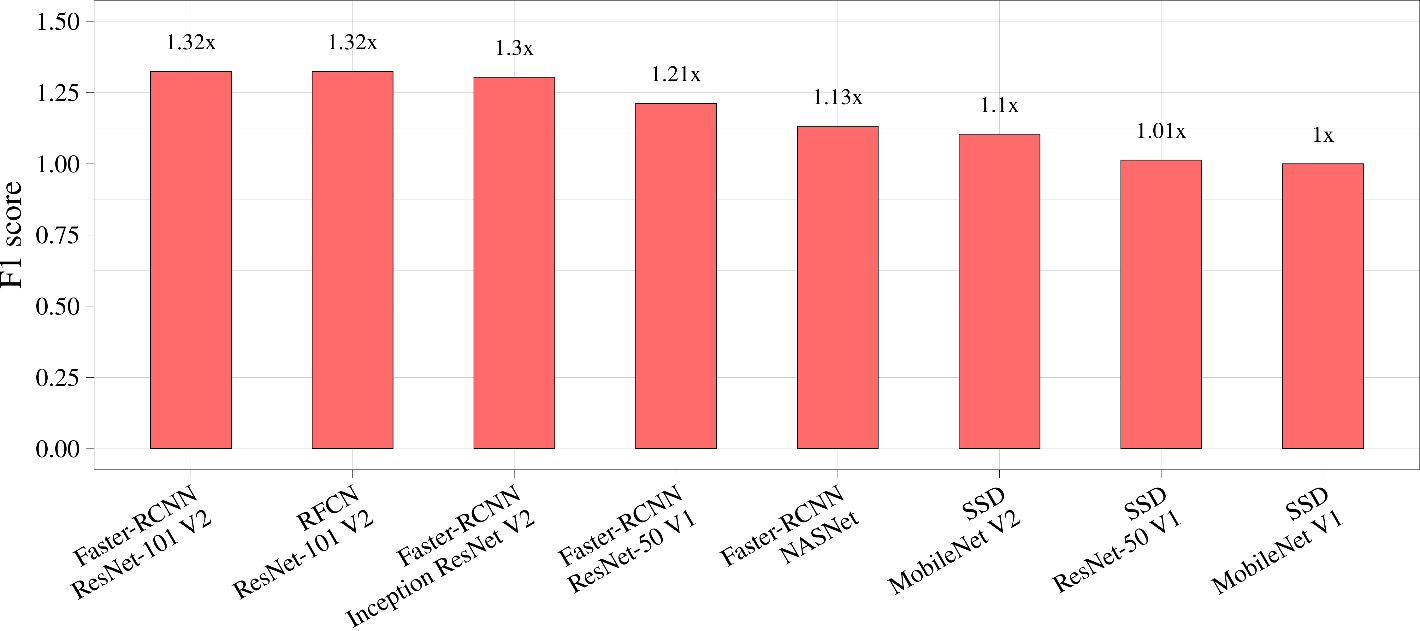 |
| (b) Relative values of F1 score |

**Appendix F. Comparison of precision of the selected neural network models**

| 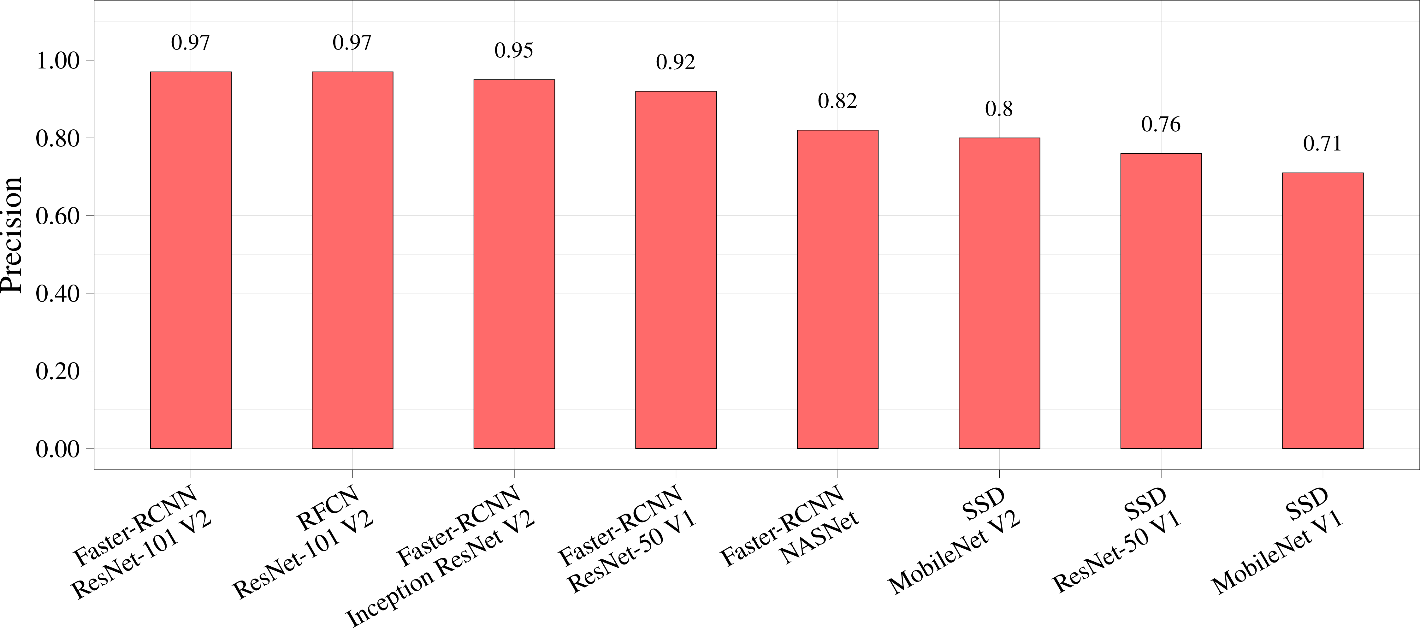 |
| --- |
| (а) Absolute values of precision |
| 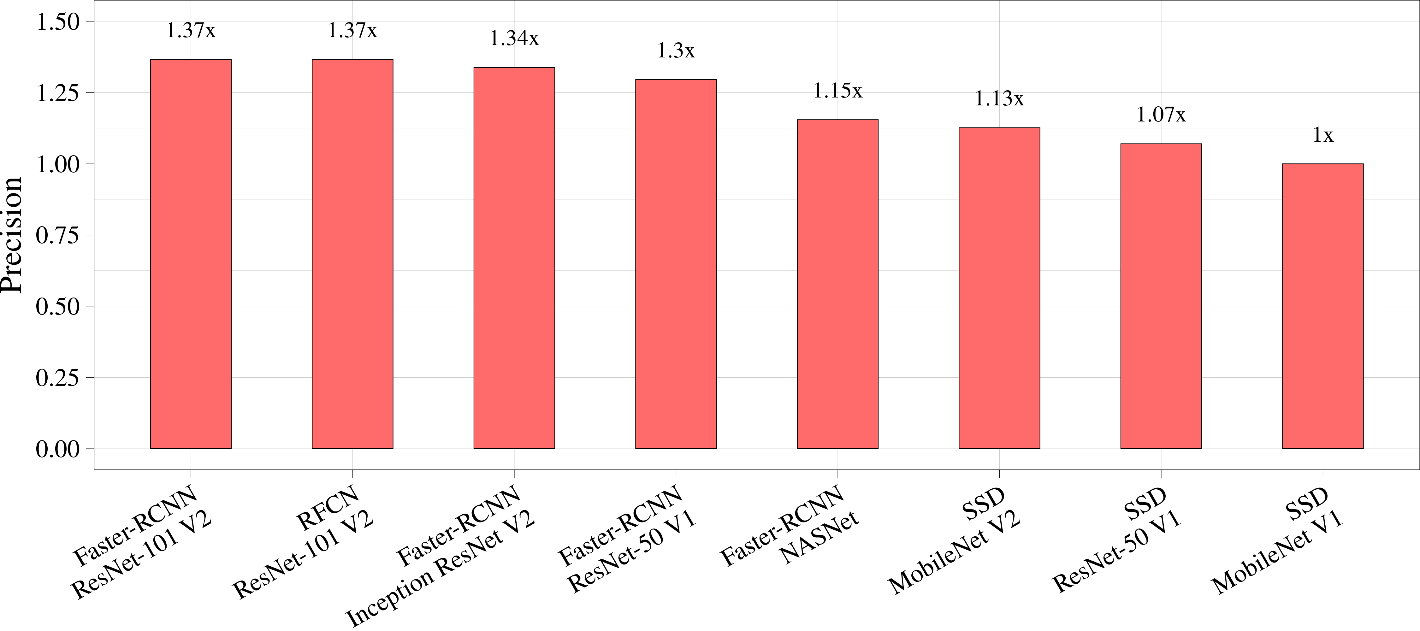 |
| (b) Relative values of precision |

**Appendix G. Comparison of recall of the selected neural network models**

| 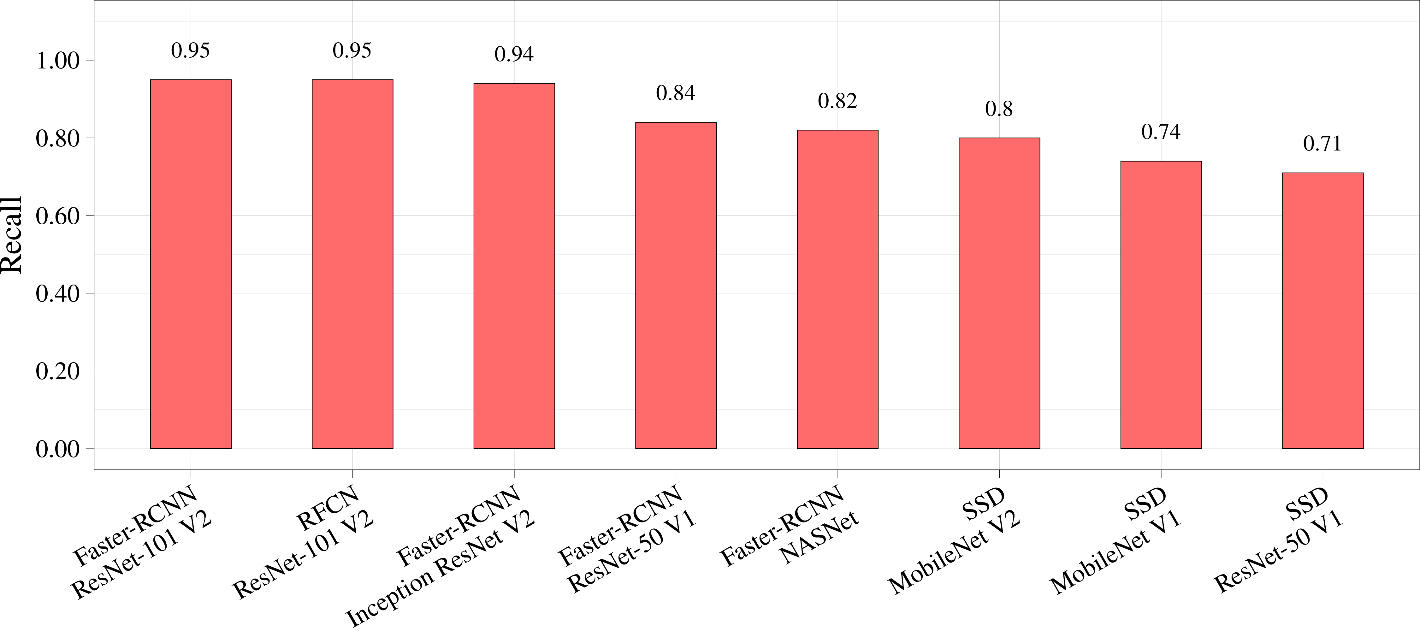 |
| --- |
| (а) Absolute values of recall |
| 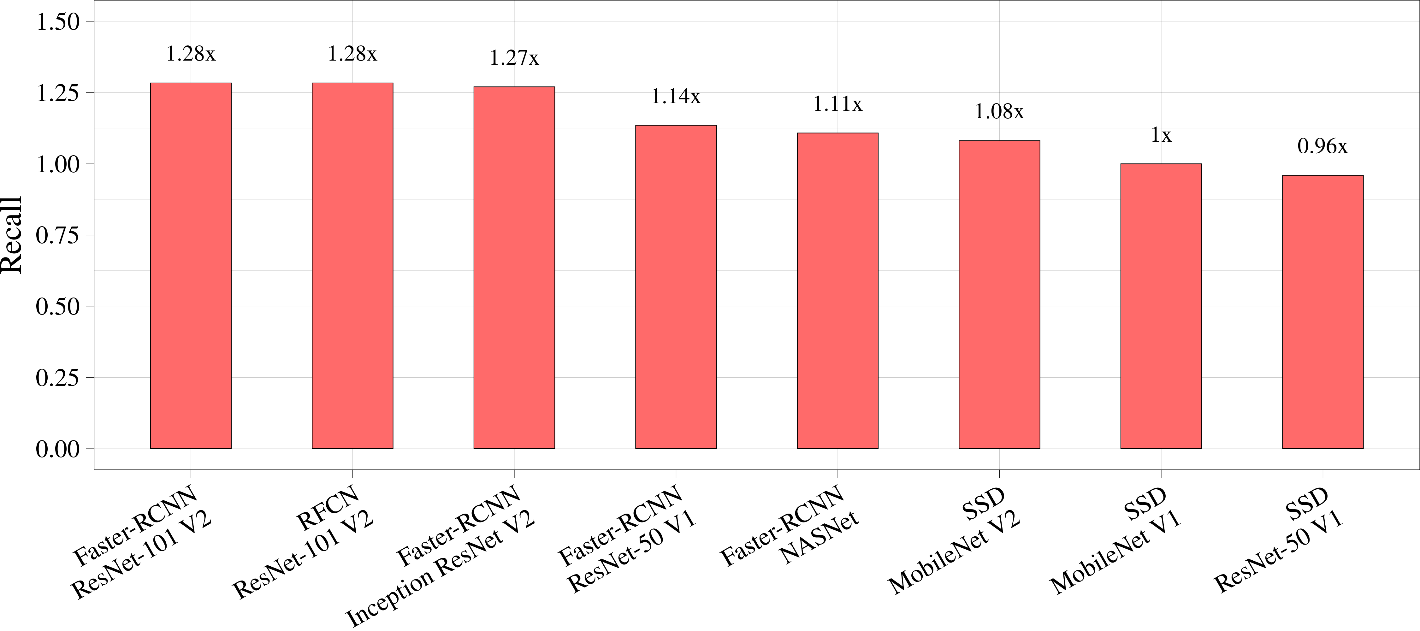 |
| (b) Relative values of recall |

**Appendix H. An example of new data prediction in patient 1 using all tested networks**

| 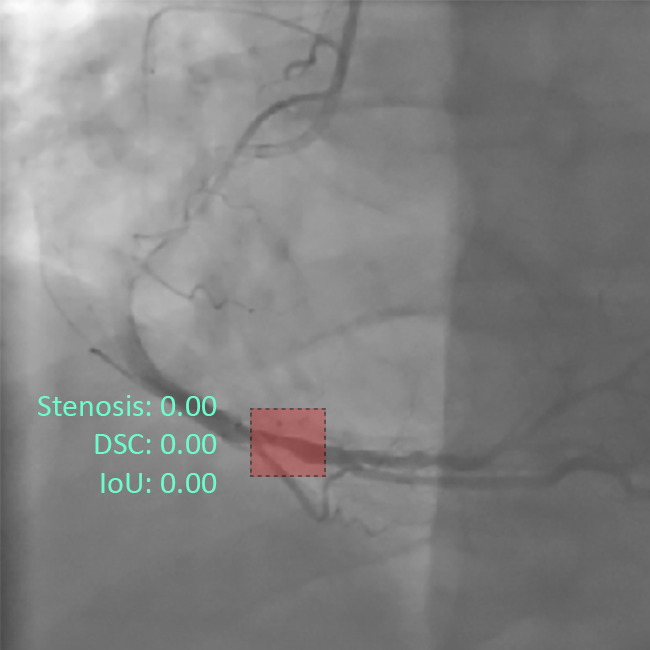 | 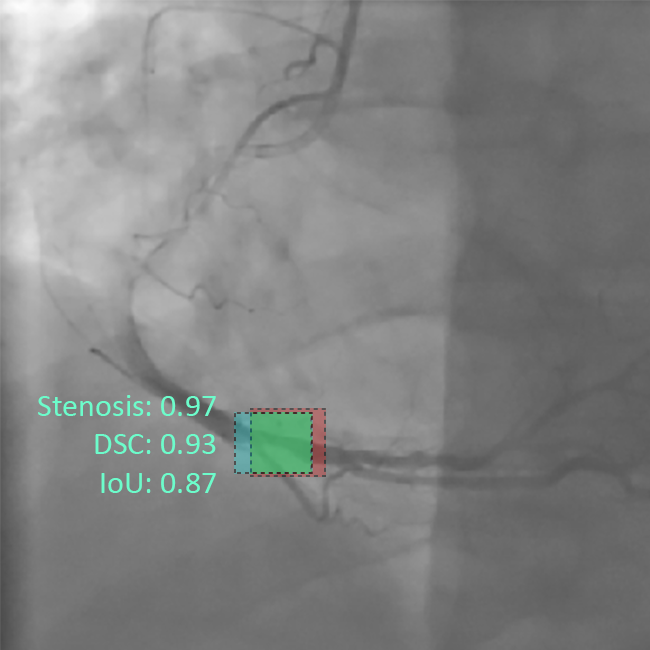 |
| --- | --- |
| (a) SSD MobileNet V1 | (b) SSD MobileNet V2 |
| 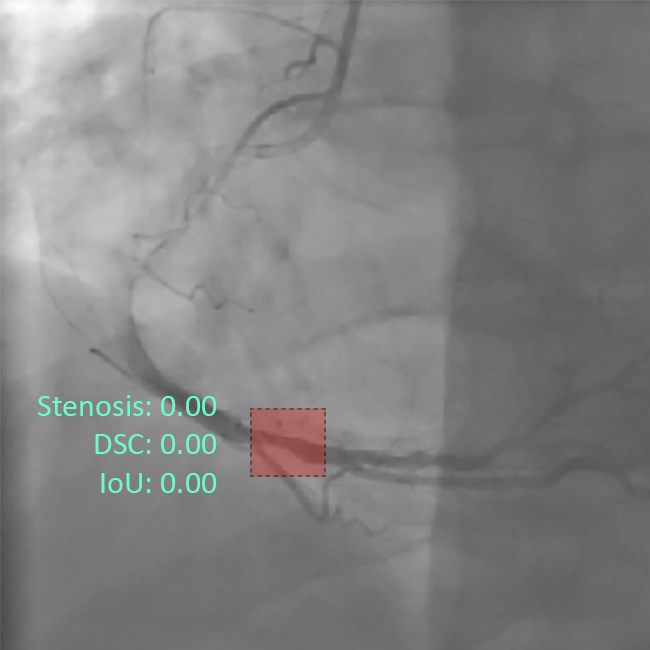 | 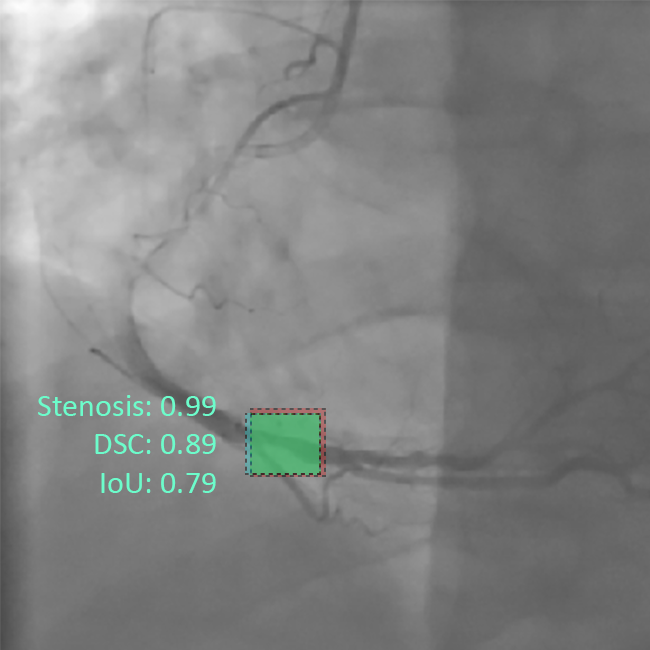 |
| (c) SSD ResNet-50 V1 | (d) Faster-RCNN ResNet-50 V1 |
| 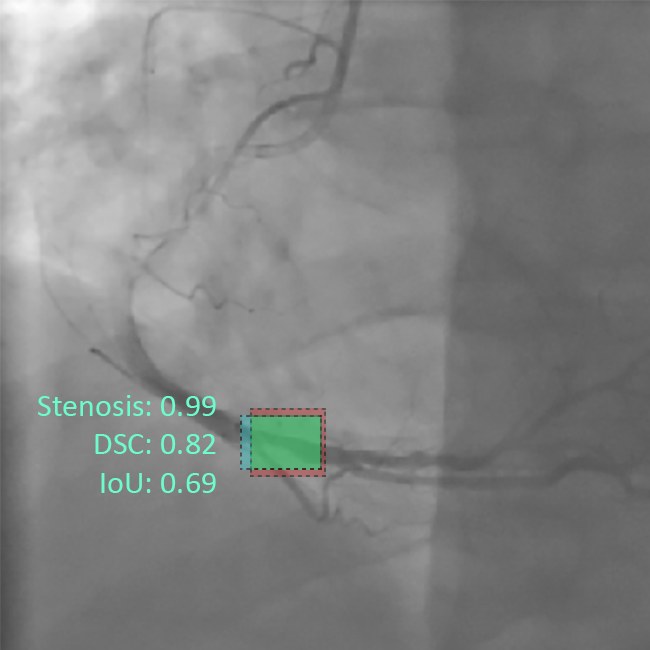 | 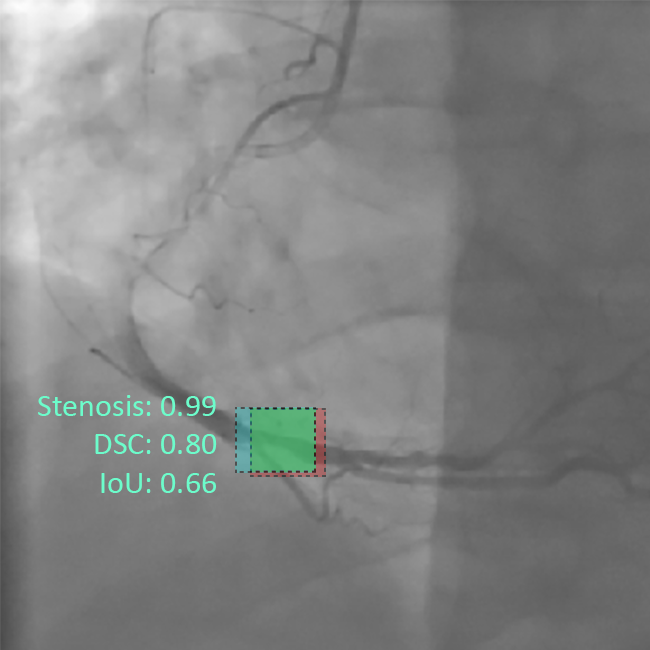 |
| (e) RFCN ResNet-101 V2 | (f) Faster-RCNN ResNet-101 V2 |
| 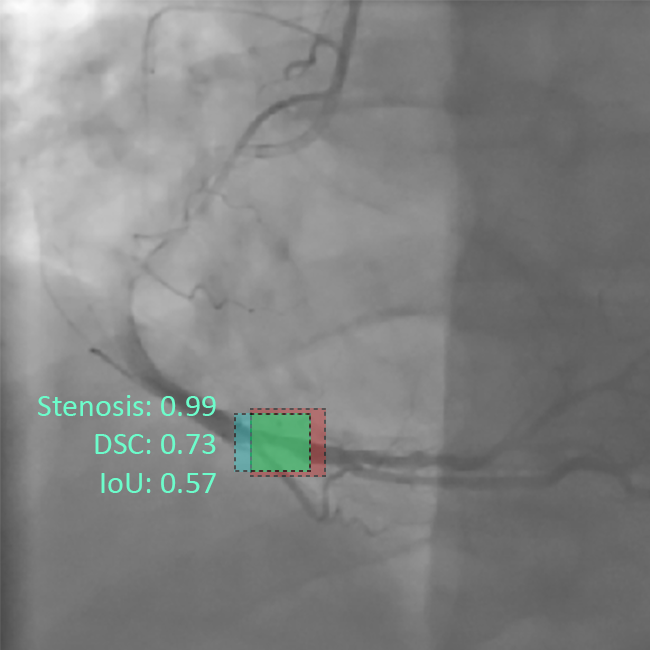 | 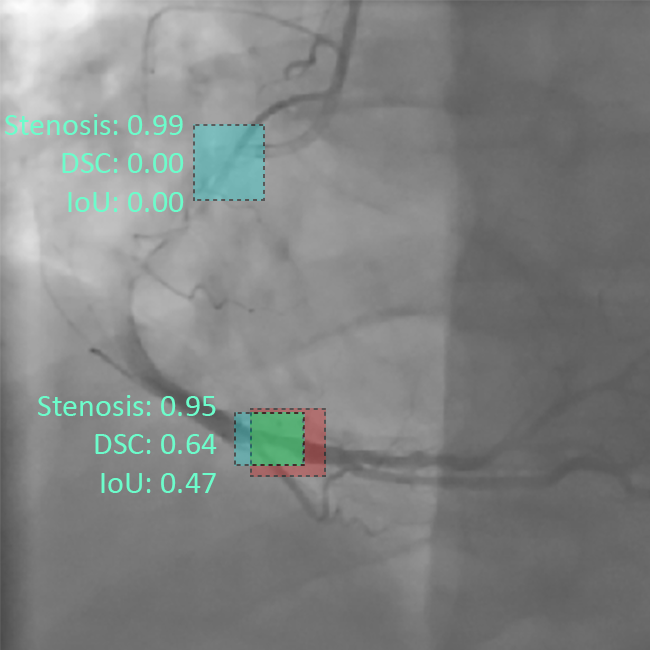 |
| (g) Faster-RCNN Inception ResNet V2 | (h) Faster-RCNN NASNet |

**Appendix I. An example of new data prediction in patient 2 using all tested networks**

| 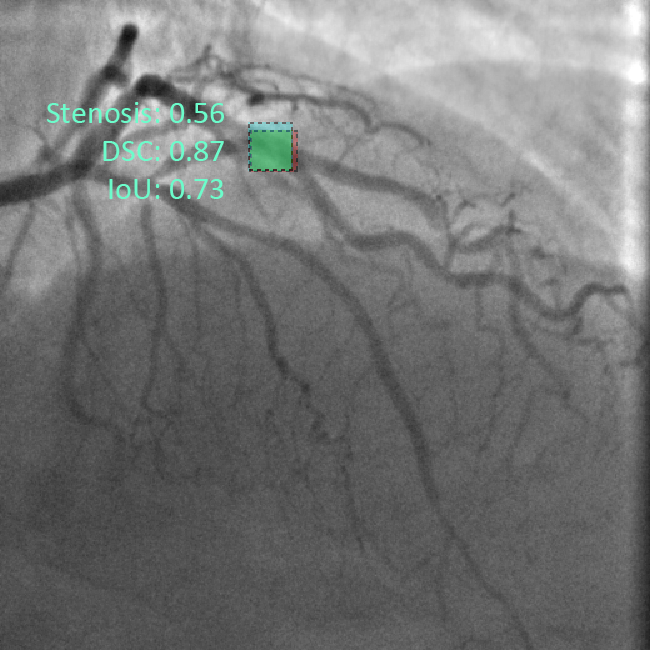 | 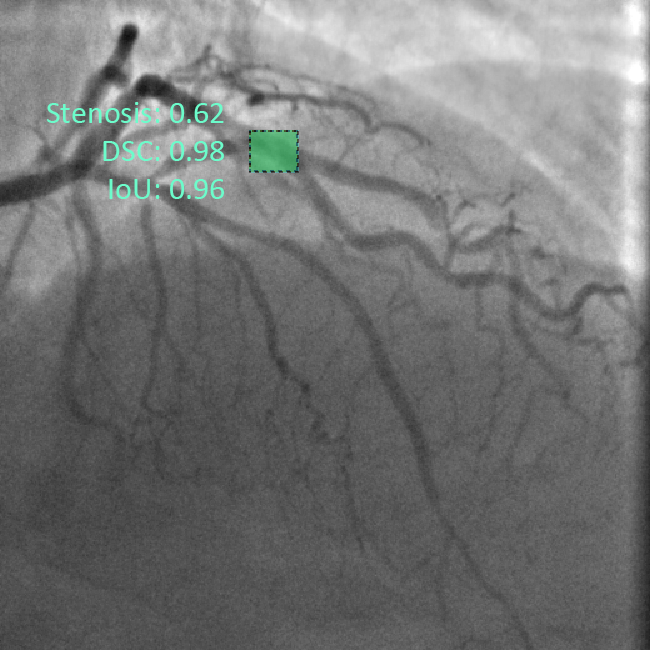 |
| --- | --- |
| (a) SSD MobileNet V1 | (b) SSD MobileNet V2 |
| 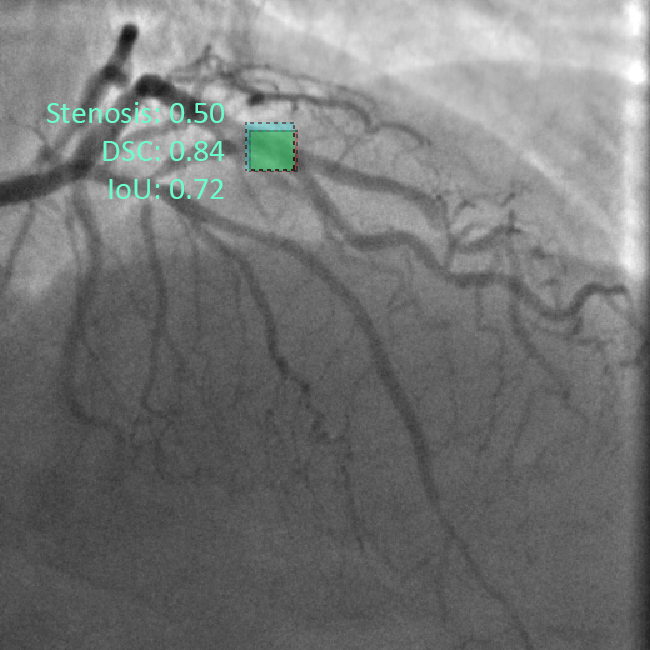 | 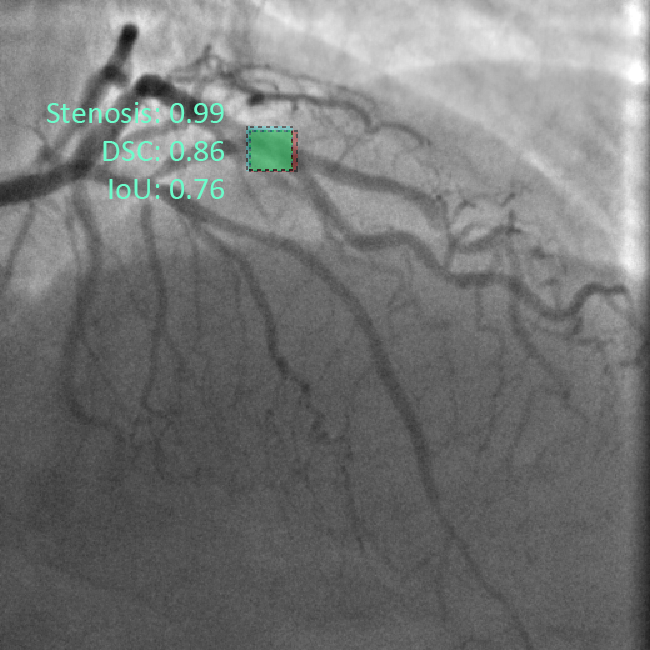 |
| (c) SSD ResNet-50 V1 | (d) Faster-RCNN ResNet-50 V1 |
| 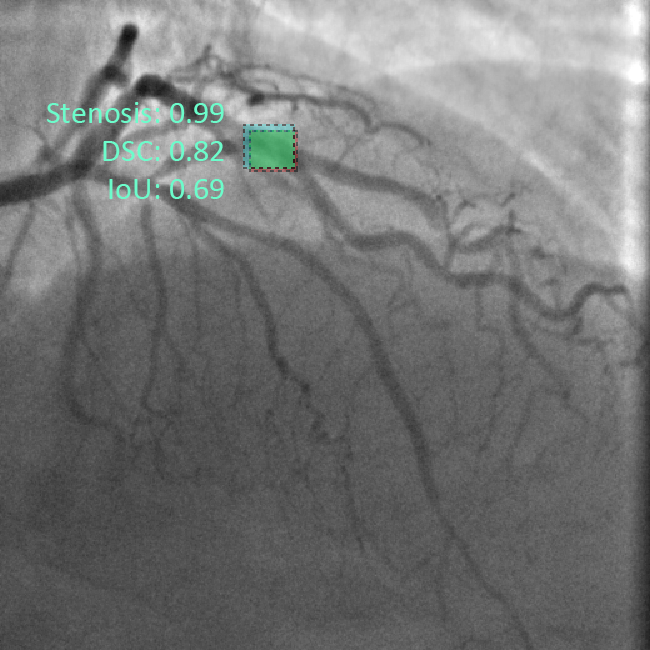 | 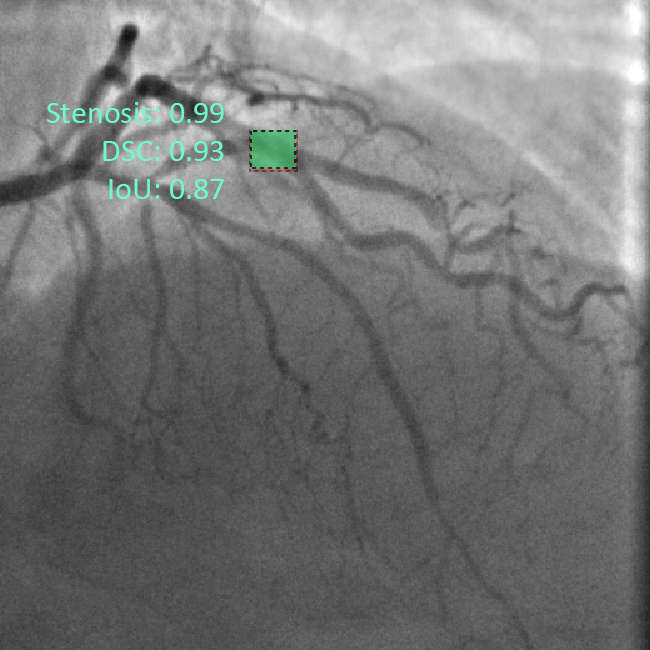 |
| (e) RFCN ResNet-101 V2 | (f) Faster-RCNN ResNet-101 V2 |
| 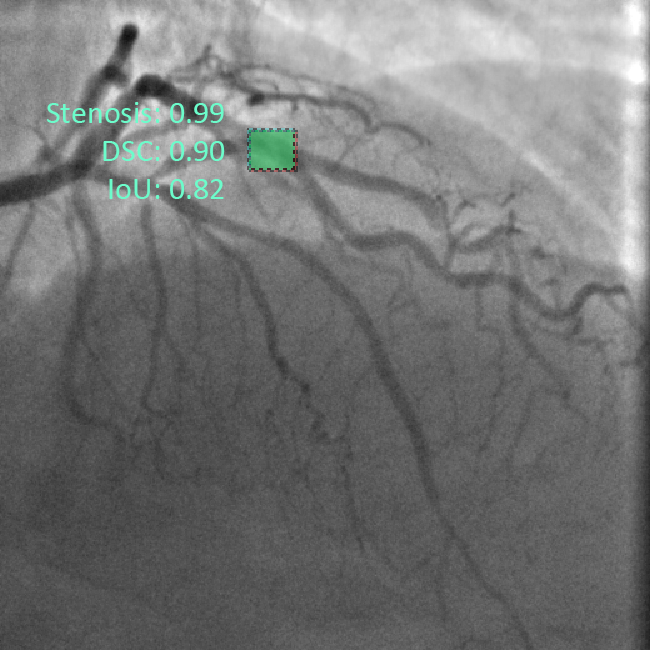 | 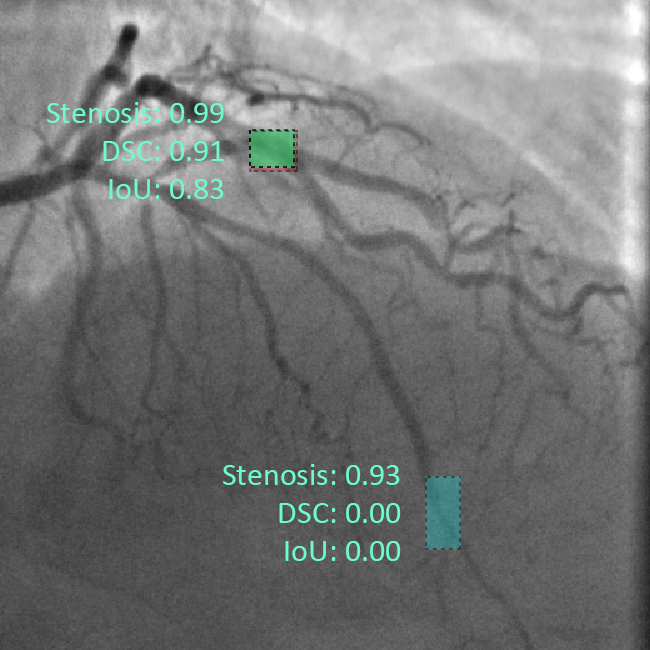 |
| (g) Faster-RCNN Inception ResNet V2 | (h) Faster-RCNN NASNet |

**Appendix J. An example of new data prediction in patient 3 using all tested networks**

| 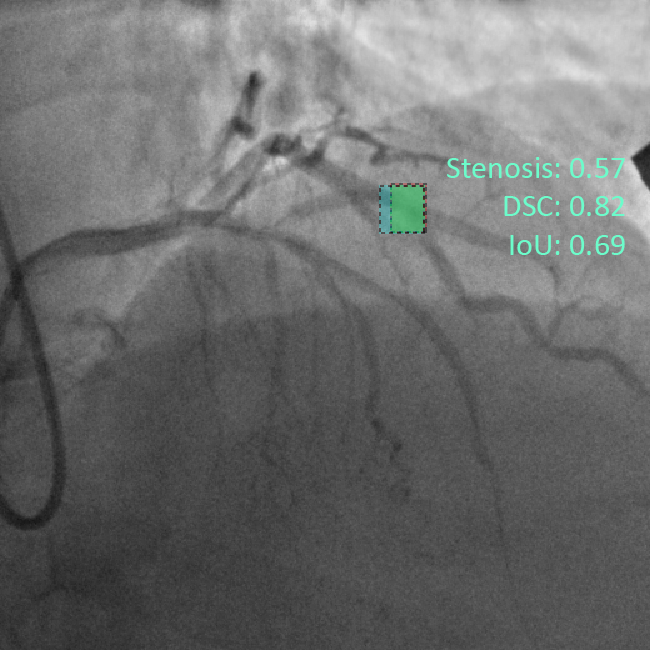 | 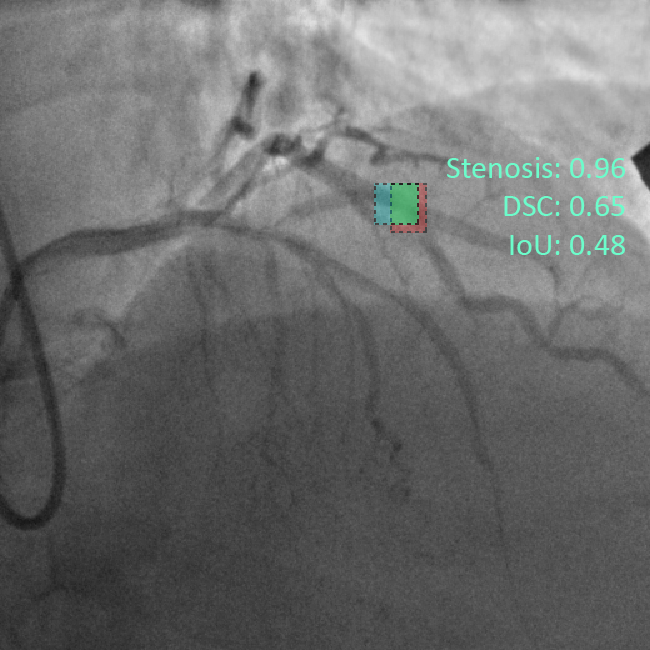 |
| --- | --- |
| (a) SSD MobileNet V1 | (b) SSD MobileNet V2 |
| 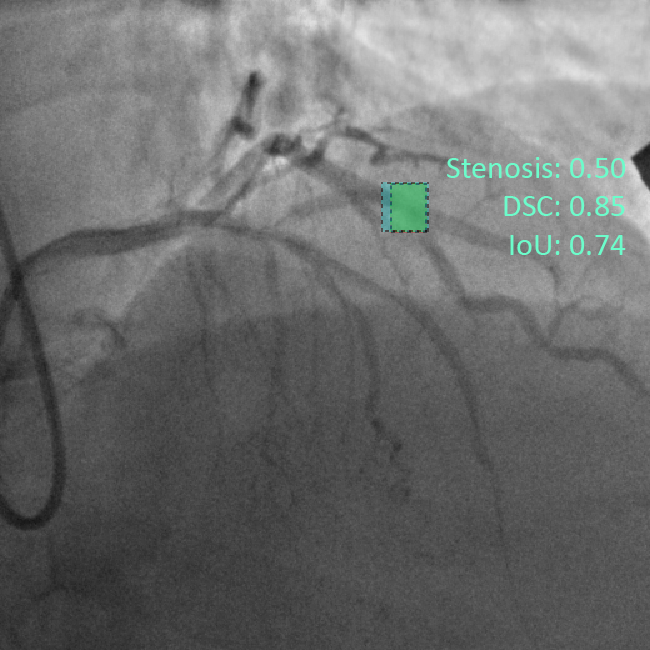 | 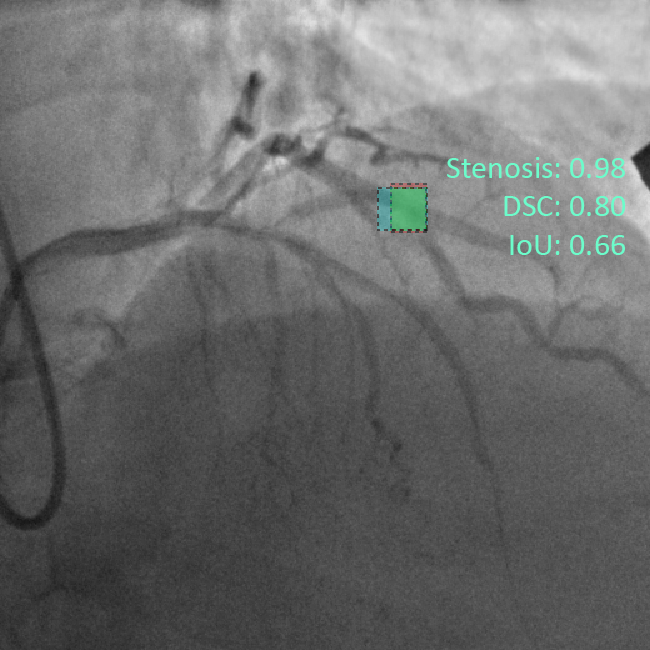 |
| (c) SSD ResNet-50 V1 | (d) Faster-RCNN ResNet-50 V1 |
| 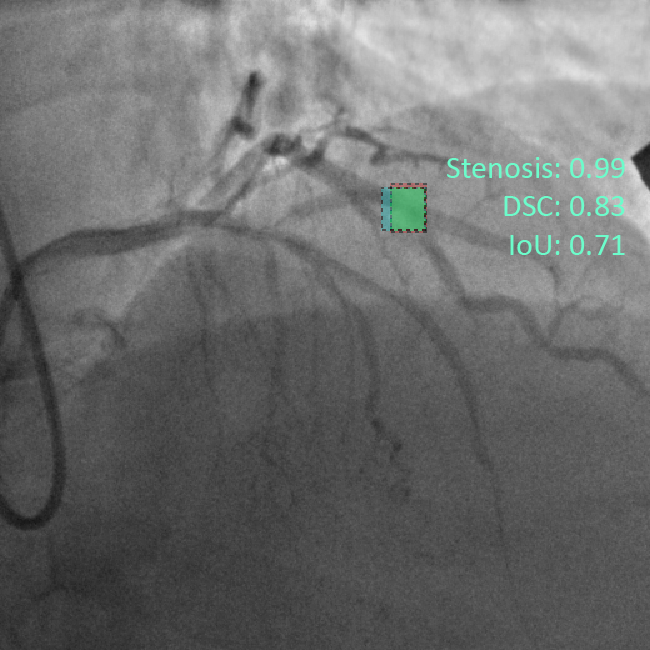 | 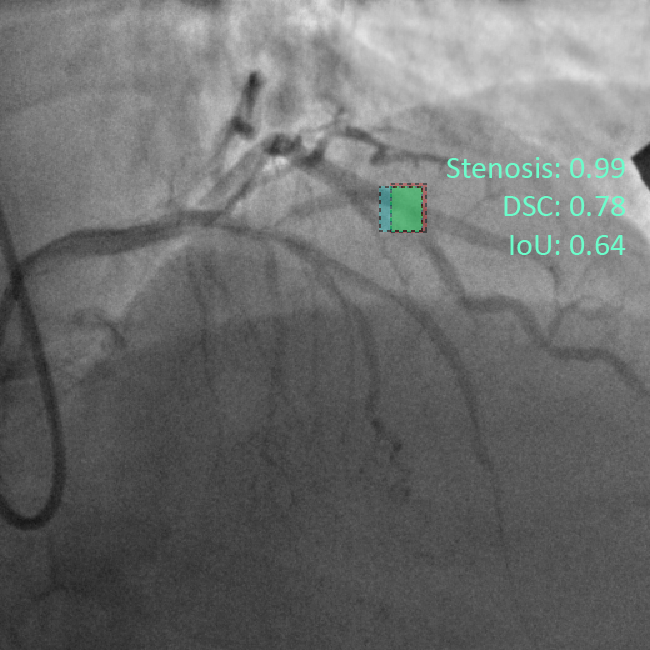 |
| (e) RFCN ResNet-101 V2 | (f) Faster-RCNN ResNet-101 V2 |
| 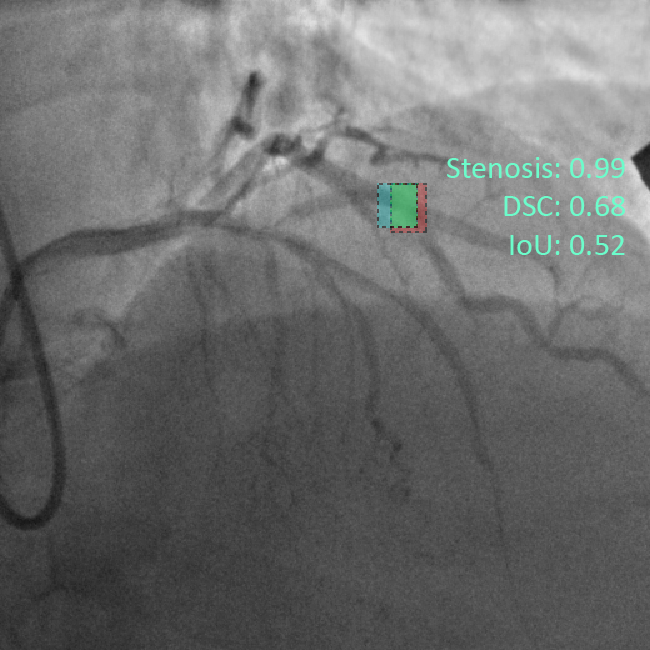 | 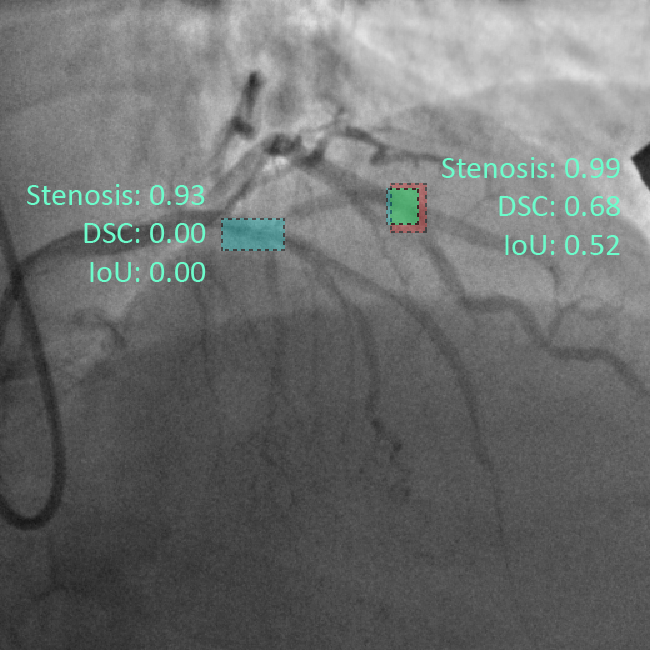 |
| (g) Faster-RCNN Inception ResNet V2 | (h) Faster-RCNN NASNet |
